# Acute exposure of microwave impairs attention process by activating microglial inflammation

**DOI:** 10.1186/s13578-023-01162-9

**Published:** 2024-01-04

**Authors:** Shaofei Jiang, Yingping Ma, Yuan Shi, Yong Zou, Zhenqi Yang, Weijia Zhi, Zhe Zhao, Wei Shen, Liping Chen, Yan Wu, Lifeng Wang, Xiangjun Hu, Haitao Wu

**Affiliations:** 1 Department of Neurobiology, Beijing Institute of Basic Medical Sciences, Beijing, China; 2 Beijing Institute of Radiation Medicine, Beijing, China; 3 School of Basic Medical Sciences, Anhui Medical University, Hefei, Anhui Province China; 4 Key Laboratory of Neuroregeneration, Co-Innovation Center of Neuroregeneration, Nantong University, Nantong, Jiangsu Province China; 5 Chinese Institute for Brain Research, Beijing, China

**Keywords:** Attention deficits, 5-CSRT, Cerebrospinal fluid, mPFC, Microglial activation, Inflammation, Microwave radiation, NMDAR, Minocycline

## Abstract

**Background:**

Attention provides the foundation for cognitions, which was shown to be affected by microwave (MW) radiation. With the ubiquitous of microwaves, public concerns regarding the impact of MW radiation on attention has hence been increased. Our study aims to investigate the potential effect and mechanism of acute microwave exposure on attention.

**Results:**

We identified obvious impairment of attention in mice by the five-choice serial reaction time (5-CSRT) task. Proteomic analysis of the cerebrospinal fluid (CSF) revealed neuroinflammation and microglial activation potentially due to acute MW exposure. Moreover, biochemical analysis further confirmed microglial activation in the prefrontal cortex (PFC) of mice subjected to acute MW exposure. Finally, minocycline, a commercially available anti-inflammatory compound, attenuated neuroinflammation, inhibited the upregulation of N-methyl-D-aspartic acid receptor (NMDAR) including NR2A and NR2B, and also accelerated the attentional recovery after MW exposure.

**Conclusions:**

We believe that microglial activation and NMDAR upregulation likely contribute to inattention induced by acute MW exposure, and we found that minocycline may be effective in preventing such process.

**Supplementary Information:**

The online version contains supplementary material available at 10.1186/s13578-023-01162-9.

## Introduction

Microwave (MW) radiation have been extensively used in our daily life. Due to the use of electronic products such as mobile phones and televisions, attention ability was found to be compromised in humans with impaired performance in divided and selective attention test as well as self-reported attention problems [1–3]. Attention is necessary for high-level cognition, and attention deficits are the hallmark of psychiatric disorders, such as attention-deficit/hyperactivity disorder [4, 5]. Prefrontal cortex (PFC) has been postulated as the key node for thought driven top-down attention with numerous evidence revealing its role using model organisms. With the studies in mice, researchers reported that lesions of the medial PFC (mPFC) increased premature responses, omissions [6] and decreased response accuracy [7] in the five-choice serial reaction time task (5-CSRT), a conventional method evaluating attention in mice. In addition, functional alteration of the activity and/or connectivity of the mPFC with its neural circuitry targets using pharmacogenetic manipulation were found to alter omissions and accuracy in the 5-CSRT for mice [8]. Therefore, it is legitimate to question whether MW induced attention inability was caused by its potential influences on the functions of the mPFC.

Previous studies regarding the mechanism of MW effects on the brain were largely focusing on the altered mitochondrial activity [9], and damaged DNA strains [10, 11]. It is also suggested that radiation induces neuroinflammation in the brain, associated with upregulated expression of cytokines such as tumor necrosis factor α (TNF-α) and interferon γ [12]. Therefore, we hypothesize that MW exposure compromises attention possibly by activating neuroinflammatory response in the brain. Minocycline, a second generational broad-spectrum antibiotic, was found to reduce the overloaded microglia and lesion size in the model of traumatic brain injury (TBI) of the rat cortex and thalamus [13], and also mitigated cognitive deficits such as impaired spatial learning and memory [13–15]. Hence, we postulated that minocycline might play a protective role in preventing MW radiation-induced attentional deficits.

Moreover, microglia, the principal immune cells that permanently reside in the central nervous system (CNS), were found to be able to communicate with neurons [16] through the activation of neuronal N-methyl-D-aspartic acid receptor (NMDAR) [17, 18]. Notably, NMDAR is one of the glutamate receptors enriched in the brain [19] playing a pivotal role in regulating PFC activity and attention [20–23]. Considering that NMDAR might contribute to the long-term cognitive impairment after MW exposure [24], the role of NMDAR in controlling attention after acute MW exposure could be further elucidated. In this study, we explored the impact and potential underlying mechanisms of MW on attention, and evaluated the effects of minocycline on MW-induced inattentional behavior in mice.

## Methods

### Animals

The male C57BL/6J mice (aged 8 weeks, weighed 22–24 g) were provided by SPF (Biotechnology Co., Ltd., Beijing, China, SCXK (Jing) 2019-0010). The mice were group-housed (four to five per cage) on a 12-h light/dark cycle (lights on from 6:30 a.m. to 6:30 p.m.) in a temperature (20–24 °C)- and humidity (50–60%)-controlled housing facility. Food was freely available throughout the study, while access to water was adjusted to stabilize each mouse at 90% of free-drinking body weight. The mice were weighed every morning before training/testing in the 5-CSRT.

### Treatment

Minocycline hydrochloride (MCE, Cat# HY-17412, New Jersey, USA) was dissolved in a 5 mg/mL warmed normal saline solution and then given at 40 mg/kg (intraperitoneal injection) [25]. Mice received minocycline or vehicle twice a day (12 h intervals) for 2 consecutive days. And mice were received MW radiation at 2 h after intraperitoneal injection of minocycline or vehicle. Then, mice were sacrificed at 1 h after MW and brain was used for protein, or morphological analyses.

### MW exposure system and dosimetry

**Fig. 1:**
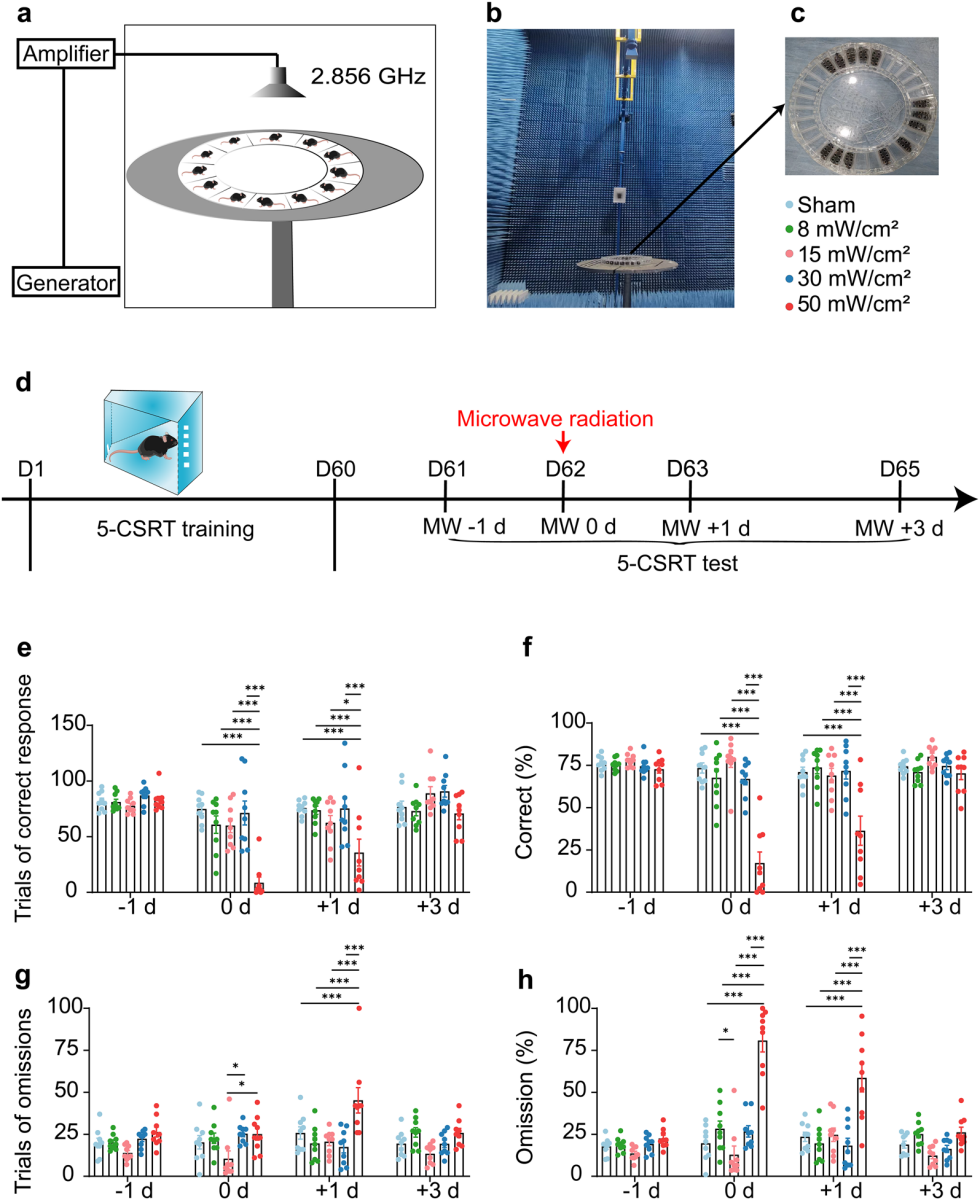
Short-term attention deficits caused by 50 mW/cm^2^ MW exposure. **a** Diagram of the microwave radiation. **b**–**c** Radiation apparatus. **d** Schematic representation of the experimental procedure. **e**–**h** Quantitative analysis of the trials of correct response, percentage of correct, trials of omission or percentage of omission in mice after MW exposure, respectively. Data are presented as mean ± SEM (n = 9). *P < 0.05, ***P < 0.001 (Two-way ANOVA and Tukey’s multiple comparisons test)

Pulsed MWs at the frequency of 2.856 GHz were generated by the MW exposure apparatus (Fig. 1a–c) [26]. MW energy was transmitted by rectangular waveguide and A16-dB standard-gain horn antenna to an electromagnet-ic shield chamber. The average power densities were measured with a waveguide antenna, the GX12M1CHP power meter (Guanghua Micro-electronics Instruments, Hefei, China) and GX12M30A power heads [27]. To explore whether MW radiation affects the attention, the well-trained mice were randomly assigned to five groups, including sham, 8, 15, 30 and 50 mW/cm^2^ group (n = 9), correspond to the average whole body special absorption rate (SAR) of 2.1, 3.9, 7.8 and13g W/kg in human, respectively. The sham group mice enter the same apparatus and exposure the similar dB noises without MW radiation. Mice were tested at 1 day before MW (-1 d), 1 h after MW exposure (0 d), 1 day after MW (+ 1 d) and 3 days after MW (+ 3 d), respectively (Fig. 1d).

### 5-CSRT

#### Apparatus

Given rodents were rather efficient in learning procedure with touchscreen interfaces compared to other traditional devices (nose-poke and press-lever) [28], we therefore selected the touch-screen version of 5-CSRT to assess the mouse attention after MW exposure. The apparatus comprised eight mouse operant chambers, each housed within a ventilated sound-attenuating box (Bussey-Saksida touch screen, Lafayette Instrument Company, Cambridge, UK). Operant chambers were controlled by a Lafayette Instruments control unit, which running ABET II Touch (Version 21.02.26.0, Lafayette Instrument Company, Cambridge, UK) and Whisker software (Cambridge University Technical Services Ltd, Cambridge, UK).

#### Task

5-CSRT training and testing were carried out as previously described [29], details in Additional file 5: Table S1. In brief, the mice were trained to respond to one of five touch-screens, that pseudo-randomly illuminated for 1 s (5 s limited hold), to earn 20 μL 2% sucrose solution. Failure to respond within the limited hold (omission responses) or incorrect response (i.e., whisker poke to wrong screen) were punished with a 2 s shine of white houselights and 5 s timeout. Each session of training phase finished after 30 min or the completion of 100 trials, whichever occurred first. While at testing phase, mice complete trials unlimitedly within 30 min. Training/testing was carried out once a day during dark phase, 6 days a week. Mouse attention is measured by these variables, including the trials of correct responses, omissions and premature, the percentage of accuracy ([correct responses/(correct + incorrect)] × 100), the percentage of correct ([correct responses/(correct + incorrect + omissions)] × 100) and the percentage of omissions ([omissions/(correct + incorrect responses + omissions)] × 100) [5, 29].

### Cerebrospinal fluid proteome analysis

#### CSF sample collection and protein extraction

Mice were randomly assigned to three groups (n = 10), including sham, MW 0 d (50 mW/cm^2^) and MW + 3 d. A micropipette was inserted into the cerebellomedullary cistern of anesthetized animal to slowly extract the mouse CSF. Approximately 5–10 μL of CSF was collected per mouse. Next, CSF were transferred to a sterile vial, stored at − 80 °C before analysis. From each CSF sample, 1 μL was taken for quality control (QC), which was injected frequently to monitor the reproducibility of the Liquid Chromatograph Mass Spectrometer (LC–MS/MS). The individual CSF samples (5 μL) and QC sample were deoxidized with 20 mM dithiothreitol for 5 min at 95 °C and carbamidomethylated with 50 mM iodoacetamide for 45 min at room temperature (22–24 °C) in dark. Subsequently, all samples were digested with trypsin (1:50) in 25 mM of NH_4_HCO_3_ buffer (pH = 8) and incubated overnight at 37 °C [30]. The average of protein number was 1213.5, 1217.3, 1156.7 and 1268 in sham, MW 0d, MW + 3 d and QC group, respectively.

#### Liquid chromatography with tandem mass spectrometry

Orbitrap Exploris 480 (Thermo Scientific) coupled with the EASY-nLC 1000 was used for analysis in the data-independent acquisition-mass spectrometry (DIA-MS) mode. The digested peptides were separated on an RP C18 self-packing capillary LC column (75 μm × 100 mm; particle size 3 μm). The eluted gradient used was 5–30% of buffer B2 (0.1% formic acid, 99.9% acetonitrile; flow rate, 0.3 μL/min), and peptides were eluted for 25 min.

For DIA analysis, a variable isolation window with 60 windows was employed for MS acquisition. According to the precursor m/z distribution of the pooled sample, the precursor ion number was equalized in each isolation window. The full scan range was set from 350 to 1200 m/z and screened at a resolution of 120,000, followed by DIA scans with a resolution of 30,000 (higher-energy C-trap dissociation (HCD) collision energy: 30%; aggrecan target: 200%; maximum injection time: 50 ms).

#### Data processing and analysis

The raw DIA data were analyzed by Spectronaut Pulsar 17.1 (Biognosys) with default settings. In brief, the retention time prediction type was set to dynamic iRT. Interference correction on the MS2 level was enabled. Peptide intensity was calculated by summing the peak areas of their respective fragment ions for MS2, and the protein intensity was calculated by summing the intensity of their respective peptides. Cross-run normalization was allowed to correct the systematic variance in the LC–MS/MS performance, and a local normalization strategy was applied. The normalization was based on the assumption that on average, a similar number of peptides was either upregulated or downregulated, and most peptides remain unaltered across runs. Protein inference was performed with the ID picker algorithm implemented in Spectronaut. All data were filtered by a Q value cutoff of 0.01 (corresponding to a flavin adenine dinucleotide of 1%). Finally, the volcanic map (wkomics.omicsolution.com) was utilized to present the differentially expressed protein changes between different groups (MW 0d vs. Sham, MW + 3 d vs. Sham group). The subcellular localization of proteins was annotated by the Ingenuity Pathway Analysis (IPA) software (Ingenuity Systems, Mountain View, CA). The significance of enrichment for each subcellular localization term was then calculated by one-sided Fisher’s exact test, using CSF proteins identified in this study as the background. Overrepresentation analysis of protein functions was performed by IPA [31].

### Open-field test

Locomotor activity was measured using an open-field apparatus (50 × 50 × 40 cm) during dark phase. Mice were individually placed in the centre of the apparatus allowing free exploration. Total distance travelled and average speed by each mouse was record for 10 min with behavior tracking software (ANY-maze 7.06 (64-bit) 1999–2021, Stoelting Co, Wood Dale, US), as described previously [32].

### Immunofluorescence staining

Immunofluorescence staining was applied to study the localization and expression of Iba-1 and TNF-α. Mice were anesthetized by 1% pentobarbital (50 mg/kg, intraperitoneal injection) (China National Pharmaceutical Group Corporation, Beijing, China) and perfused with saline first, followed by pre-cooled 4% paraformaldehyde. Then, the mouse brain was isolated and postfixed in 4% paraformaldehyde overnight. Dehydration was applied with 15% and then 30% sucrose solutions. Coronal slices of the mouse brain (35 μm thick) were obtained by utilizing frozen microtome (Thermo Fisher Scientific, Waltham, MA, USA). As for staining, the brain slices were blocked with 3% bovine serum albumin in phosphate-buffered saline (Solarbio, Beijing, China) and 0.3% Triton X-100 for 1.5 h. The primary antibodies were incubated overnight at 4 °C: goat anti-Iba-1 (1:500, Abcam, Cat# ab5076, RRID: AB_2224402, Cambridge, UK), and mouse anti-TNFα (1:200, Abcam, Cat# 1793, RRID: AB_302615). Secondary antibodies were then incubated at 20–24 °C for 1.5 h: Alexa Fluor 488-conjugated donkey anti-goat IgG (1:500, Biotium, Cat# 20016, RRID: AB_10563028, CA, USA) and Alexa Fluor 568-conjugated donkey anti-mouse IgG (1:500, Biotium, Cat# 20105, RRID: AB_10557030). Nuclei were counterstained with 4ʹ,6-diamidino-2-phenylindole (Yangguang Bio, Cat# C190401). All images were captured on an Olympus FV-1200 confocal microscope (Olympus, PA, USA) and Imaris 9.3.1 software (Abingdon, Oxfordshire, UK) were used to calculate the positive rate.

### Three-dimensional reconstruction of microglia

Confocal images were captured using an Olympus FV-1200 confocal microscope (Olympus, PA, USA) and a 60 × 7 oil immersion objective. Z-stack images with 0.5-μm intervals were captured at a depth of 35 μm. Images were further analyzed for the process length, the number of process branches and terminals, as well as cell body size of microglia by using Imaris 9.3.1 software (Abingdon, Oxfordshire, UK).

### Western blot analysis

Western blot was carried out to identify protein changes of microglial activation and NMDAR (NR2A and NR2B). Total protein was extracted from mice brain tissues with lysis buffer (Sigma-Aldrich, Cat# 89,900). Protein concentration was measured by using bicinchoninic acid protein assay kit (Sigma-Aldrich, Cat# 23227). The proteins were isolated using sodium dodecyl sulfate–polyacrylamide gel electrophoresis and then transferred to polyvinylidene fluoride membranes (Yangguang Bio, Beijing, China). Membrans were then blocked with 5% bovine serum albumin and incubated overnight with primary antibodies at 4 °C. The primary antibodies for Western blot analysis were listed as follows: rabbit anti-CD36 (1:1000, ABclonal, Cat# A5792, RRID: AB_2766544), rabbit anti-PPT1 (1:1000, ABclonal, Cat# 14769, RRID: AB_2761645), rabbit anti-LRP1 (1:1000, ABclonal, Cat# A0633, RRID: AB_2861470), goat anti-Iba-1 (1:3000, Abcam, Cat# ab5076, RRID: AB_2224402), mouse anti-TNF-α (1:3000, Abcam, Cat# ab1793, RRID: AB_302615), mouse anti-Glyceraldehyde-3-phosphate dehydrogenase (GAPDH) (1:5000, Abcam, Cat# ab8245, RRID: AB_2107448), rabbit anti-CD68 (1:3000, ABclonal, Cat# A20803, RRID: AB_2940793, Wuhan, China), rabbit anti-NR2A (1:3000, ABclonal, Cat# A19089, RRID: AB_2862581), and rabbit anti-NR2B (1:3000, ABclonal, Cat# A3056, RRID: AB_2764860). The membranes were then incubated at 20–24 °C for 1.5 h with horseradish peroxidase-conjugated goat anti-mouse IgG (1:5000, ZSGB-BIO, Cat# ZB-2305, RRID: AB_2747415, Beijing, China), horseradish peroxidase-conjugated goat anti-rabbit IgG (1:5000, ZSGB-BIO, Cat# ZB-2301, RRID: AB_2747412) or horseradish peroxidase-conjugated rabbit anti-goat IgG (1:5000, ABclonal, Cat# AS029, RRID: AB_2769859). The signals were captured using chemiluminescent imaging system (SAGECREATION, Beijing, China), light intensity was quantified using ImageJ 2.0.0 software (National Institutes of Health, MD, USA), and protein expression ration was normalized by GAPDH.

### Statistical analysis

The investigator was blind to the group allocation during the experiment and data collection. The data are presented as mean ± standard error of mean (SEM). *P* < 0.05 was regarded as statistically different. One-way analysis of variance was used for analyzing the effect of MW on microglial inflammation (Fig. 3, Additional file 3: Fig. S3 and Additional file 4: Fig. S4). Two-way analysis of variance (two way-ANOVAs) was used for analyzing datasets with two groups as indicated in the results (Fig. 1, MW × time; Fig. 4, pretreatment × MW; Fig. 6, pretreatment × MW; Additional file 1: Fig. S1 b, c, MW × time; Additional file 2: Fig. S2 a and c, MW × time). Three-way ANOVAs was used for analyzing whether minocycline-pretreatment ameliorates MW-induced inattention (Fig. 5, MW × pretreatment × time). Tukey’s and Sidak’s multiple comparisons test were used for post-hoc analysis. All data were statistically analyzed using GraphPad Prism 8.0.2 (GraphPad, San Diego, CA, USA, www.graphpad.com). The figure of ‘Graphical abstract’ was made by Figdraw (ID: OSAUYcf31a, China, www.figdraw.com), others were created with Adobe IIIustrator 2020 (Adobe, USA).

## Results

### Acute MW exposure causes short-term attention deficits

At first, Two-way ANOVAs (time × MW) showed acute MW exposure impair attentional behaviors among 5-CSRT, including the trials of correct response (interaction effect, F_(12, 160)_ = 4.408, *P* < 0.0001; the main effect of time, F_(3, 160)_ = 21.88, *P* < 0.0001; the main effect of MW, F_(4, 160)_ = 15.92, *P* < 0.0001), the percentage of correct (interaction effect, F_(12, 160)_ = 8.967, *P* < 0.0001; the main effect of time, F_(3, 160)_ = 17.95, *P* < 0.0001; the main effect of MW, F_(4, 160)_ = 33.39, *P* < 0.0001), the trials of omissions (interaction effect, F_(12, 160)_ = 2.562, *P* = 0.0040; the main effect of time, F_(3, 160)_ = 2.886, *P* = 0.0375; the main effect of MW, F_(4, 160)_ = 11.98, *P* < 0.0001), and the percentage of omissions (interaction effect, F_(12, 160)_ = 10.36, *P* < 0.0001; the main effect of time, F_(3, 160)_ = 19.04, *P* < 0.0001; the main effect of MW, F_(4, 160)_ = 44.02, *P* < 0.0001) (n = 9, Fig. 1e–h). But, there was no difference in the percent of accuracy (interaction effect, F_(12, 160)_ = 1.191, *P* = 0.2937; the main effect of time, F_(3, 160)_ = 1.429, *P* = 0.2362; the main effect of MW, F_(4, 160)_ = 1.547, *P* = 0.1912) (n = 9, Additional file 2: Fig. S2a), suggesting that MW exposure likley affect the aspect of attention more than learning and cognition. Then conduct post hoc testing to further analysed when MW exposure specifically affects attention. Mice exposed to 50 mW/cm^2^ MW displayed smaller number in the trials of correct response (compared with sham, 8, 15 or 30 mW/cm^2^ group, *P* < 0.0001; Fig. 1e) and percentage of correct (compared with other four groups, *P* < 0.0001; Fig. 1f), while the percentage of omission (compared with sham, *P* = 0.0005; 8, 15 or 30 mW/cm^2^, *P* < 0.0001; Fig. 1h) was found greater at MW 0 d, suggesting 50 mW/cm^2^ MW exposure impaired attention. We noted that even a day after MW, this impairment of attention remains existed in 50 mW/cm^2^ group, including lower trial numbers of correct responses (*P* < 0.0001; 8 mW/cm^2^, *P* = 0.0002; 15 mW/cm^2^, *P* = 0.0205; 30 mW/cm^2^, *P* = 0.0001; Fig. 1e) and percentage of correct response (compared with other four groups, *P* < 0.0001; Fig. 1f), as well as higher trials of omissions (sham, *P* = 0.0005; 8, 15 or 30 mW/cm^2^, *P* < 0.0001; Fig. 1g) and percentage of omissions (*P* < 0.0001; Fig. 1h) compared to other groups. However, mice spontaneously recovered their attention behavior at 3 days after MW (*P* > 0.05; Fig. 1e–h). In addition, there were no statistical difference between sham and other densities groups (except 50 mW/cm^2^), respectively (*P* > 0.05; Fig. 1e–h). Therefore, 50 mW/cm^2^ MW exposure induced short-term attention deficits.

Moreover, it was found that MW radiation can affect the impulsivity of mice through the trials of premature (interaction effect, F_(12, 160)_ = 2.456, *P* = 0.0058; the main effect of time, F_(3, 160)_ = 1.721, *P* = 0.1648; the main effect of MW, F_(4, 160)_ = 3.386, *P* = 0.0109) in 5-CSRT (n = 9). Especially in MW 0 d, the 50 mW/cm^2^ group showed lower impulsivity than sham (*P* = 0.0389) and 15 mW/cm^2^ group (*P* < 0.0001), while the 15 mW/cm^2^ group showed higher impulsivity than 8 (*P* = 0.0104), 30 (*P* = 0.0073) and 50 mW/cm^2^ (*P* < 0.0001) group (Additional file 2: Fig. S2c).

In addition, 50 mW/cm^2^ radiation did not significantly affect the locomotor activity of mice through open-field test (n = 12), such as total distance (interaction effect, F_(2, 44)_ = 2.447, *P* = 0.0982; the main effect of time, F_(2, 44)_ = 0.4317, *P* = 0.6521; the main effect of MW, F_(1, 22)_ = 1.211, *P* = 0.2831) and average speed (interaction effect, F_(2, 44)_ = 2.521, *P* = 0.0919; the main effect of time, F_(2, 44)_ = 0.3949, *P* = 0.6552; the main effect of MW, F_(1, 22)_ = 1.256, *P* = 0.2745) (Additional file 1: Fig. S1b, c). Also, we found that acute single MW displayed a trend of anxiety-like phenotypes with less time spent in center during open field test (Additional file 6: Fig. S5b–e), but did not result in depressive-like behaviors (Additional file 6: Fig. S5f). Thus, the average power intensity of 50 mW/cm^2^ were selected to further explore the mechanism of MW alters attention.

### MW exposure promotes microglial activation and neuroinflammation

**Fig. 2:**
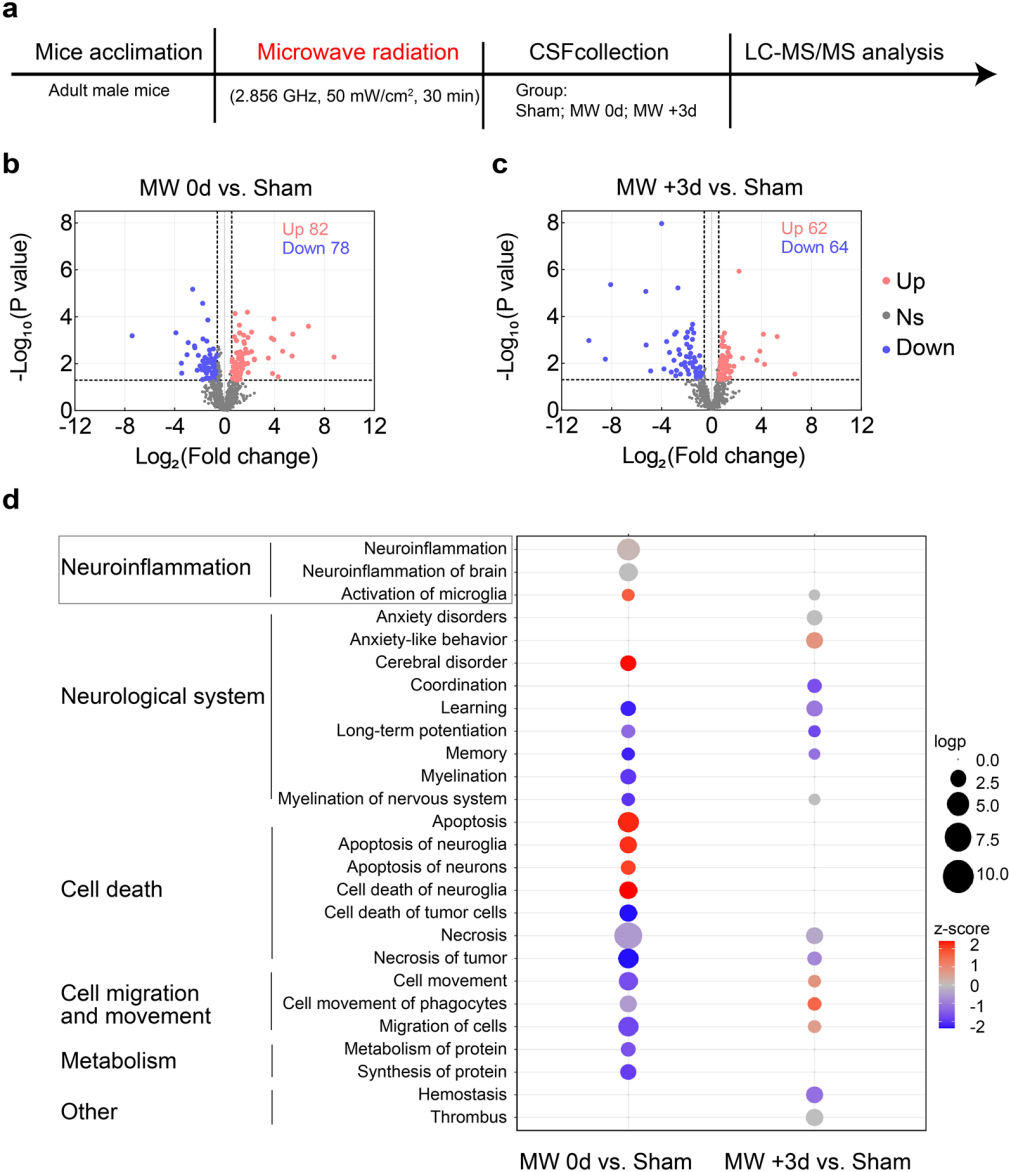
Mouse CSF proteome analysis after acute MW exposure. **a** Schematic workflow of quantitative proteomic analysis (n = 10). **b** Volcano plot for differential expressed proteins in CSF samples of MW 0 d group compared with sham. **c** Volcano plot for differential expressed proteins in CSF samples of MW + 3 d group compared with sham. Differential expressed proteins evaluation with fold change > 1.5,* P* < 0.05. **d** Evaluation of the effects of potential influencing factors on CSF proteome

Characterization of murine CSF proteomes offers key insight into the status of the CNS in animal models [30]. With aim to reveal potential biomarkers closely associated with MW exposure, LS-MS/MS analysis was applied and revealed that mice with acute MW exposure upregulated 82 proteins and downregulated 78 proteins compared to the sham mice. Three days later, MW-exposed mice (MW + 3 d group) showed 62 upregulated proteins and 64 downregulated proteins compared to sham group (Fig. 2b, c). IPA analysis revealed that MW exposure mainly affects functional pathways involving the elevation of cell apoptosis in both neurons and neuroglia, consistent with previous reports [33, 34], as well as the activation of microglia and neuroinflammation (Fig. 2d). Raw data of proteomic analysis were displayed in supplemental materials (Excel 1, 2). Noted that the activation of microglia and neuroinflammation are specifically manifested in the acute phase of irradiation, and were alleviated three days after MW exposure. In addition, results of further comprehensive analyses and experimental validations showed that the expression of CD36 was increased, whereas LRP1 and PPT1 were decreased in MW 0d group, in line with our proteomic data from CSF (Additional file 7: Fig S6 and Additional file 8: Fig. S7). These results suggest a unique role of microglia activation closely correlated with the onset of MW exposure.

**Fig. 3:**
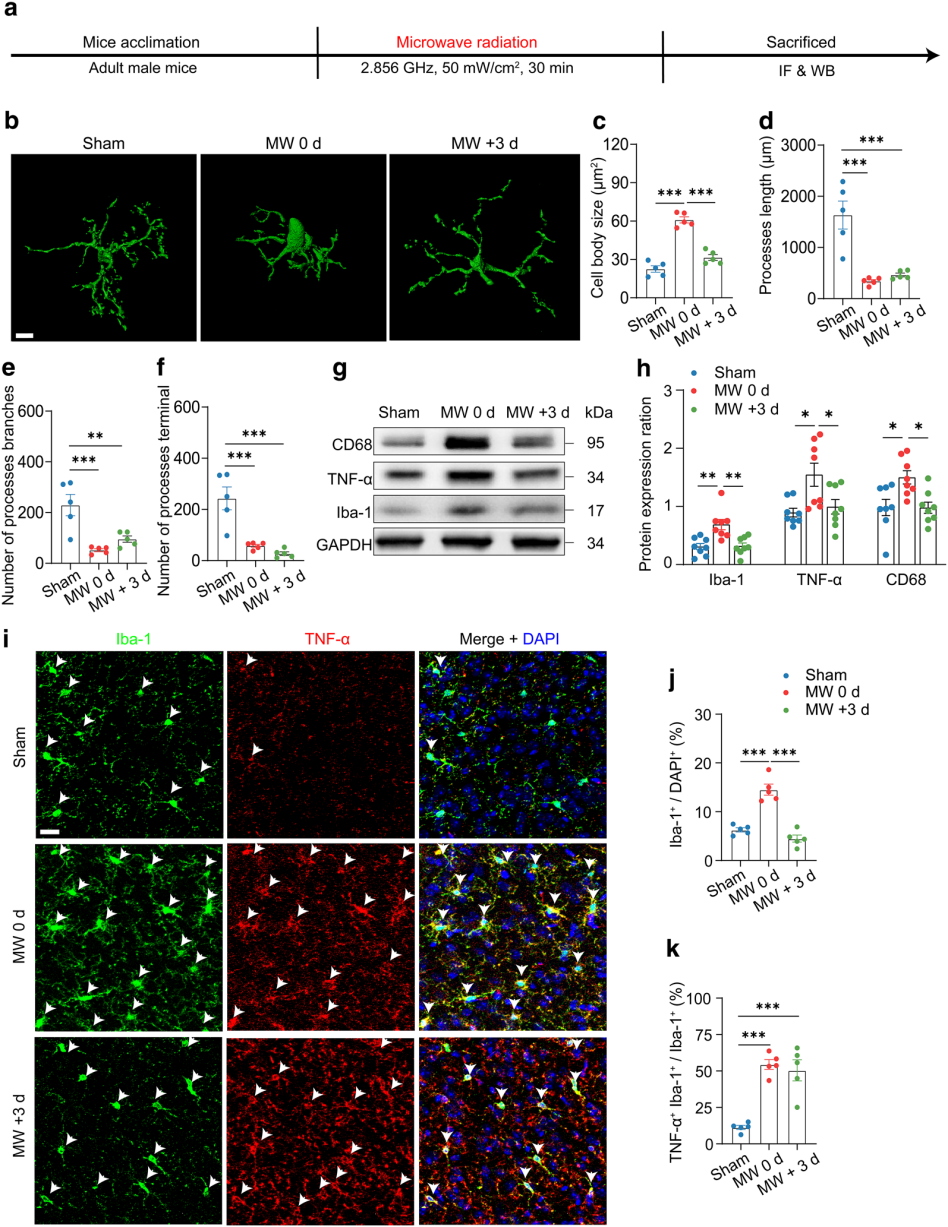
Microglial activation induced by MW exposure. **a** Timeline of experiment procedure. **b** Imaris-based 3D reconstruction images of Iba-1^+^ microglia in mPFC. Scale bars: 8 μm. **c**–**f** Imaris-based morphometric analysis of microglia in mPFC (n = 5). **g**–**h** Representative blot and quantitative analysis of the protein expression of Iba-1, TNF-α and CD68 in mPFC (n = 8). **i** Representative immunostaining of Iba-1 (green, Alexa Fluor 488) and TNFα (red, Alexa Fluor 568) in mPFC (n = 5). Scale bars: 20 μm. **j** Quantification of Iba-1 positive rate. **k** Quantification of the percentage of Iba-1^+^ and TNFα^+^ to Iba-1^+^ neurons after MW radiation. Data are presented as mean ± SEM. *P < 0.05, **P < 0.01, ***P < 0.001 (One-way ANOVA and Tukey’s multiple comparisons test)

Therefore, we hypothesize that microglial inflammation may be the mechanistic cause for both the behavioural and molecular alterations we observed. Given the function of microglia is closely related to its morphology [35], we evaluated microglial morphology in mPFC of mice after MW exposure using 3D reconstruction analysis (Imaris software) (Fig. 3b). And the results of one-way ANOVA analysis revealed difference in morphology after MW exposure (n = 5), mainly in cell body size (F_(2,12)_ = 70.33, *P* < 0.0001), processes length (F_(2,12)_ = 19.86, *P* = 0.0002), number of processes branches (F_(2,12)_ = 13.13, *P* = 0.0010) and processes terminal (F_(2,12)_ = 19.50, *P* = 0.0002). Further multiple comparisons showed enlarged cell bodies in mice with acute MW exposure compared to sham (*P* < 0.0001; Fig. 3c), accompanied by reduced processes length (*P* = 0.0003; Fig. 3d), decreased number of processes branches (*P* = 0.0010; Fig. 3e) as well as dendritic terminal (*P* = 0.0009; Fig. 3f). Besides, 3 days later, the cell bodies of microglia in mice with MW exposure (MW + 3 d group) returned to normal (compared to sham, *P* = 0.0645; MW 0 d, *P* < 0.0001), while processes length (*P* = 0.0006), number of processes branches (*P* = 0.0077) and dendritic terminal (*P* = 0.0002) were still decreased compared to sham group (Fig. 3c–f).

Furthermore, one-way ANOVA analysis of protein expression showed similar abnormal changes in Iba-1 (F_(2,21)_ = 10.30, *P* = 0.0008), TNF-α (F_(2,21)_ = 6.061, *P* = 0.0084) and CD68 (F_(2,21)_ = 6.503, *P* = 0.0063) (n = 8). And multiple comparisons revealed that these changes were increased in acute MW-exposed mice compared to sham (Iba-1, *P* = 0.0019; TNF-α, *P* = 0.0107; CD68, *P* = 0.0139, and decreased after 3 days (Iba-1, *P* = 0.0024; TNF-α, *P* = 0.0316; CD68, *P* = 0.0134; Fig. 3g, h).

Moreover, acute MW exposure led to an elevation in the positive rate of Iba-1 cells in mPFC (*P* < 0.0001; Fig. 3i, j) compared to sham and MW + 3 d, and a specific increase in the number of Iba-1^+^ and TNF-α^+^ double positive cells than sham mice (*P* < 0.0001; Fig. 3i–k). In addition, increased Iba-1^+^ was also found in other brain regions related to attention and cognition in addition to mPFC in mice exposed to MW (n = 5), including claustrum (Bregma + 1.42 to  + 1.70 mm) (F_(2,12)_ = 28.58, *P* < 0.0001), striatum (Bregma + 1.42 to  + 1.70 mm) (F_(2,12)_ = 50.00, *P* < 0.0001), nucleus accumbens (Bregma + 1.42 to  + 1.70 mm) (F_(2,12)_ = 47.26, *P* < 0.0001) and hippocampus (Bregma − 1.58 to − 1.82 mm) (F_(2,12)_ = 10.12, *P* = 0.0001), compared to sham mice (claustrum, striatum and nucleus accumbens, *P* < 0.0001; hippocampus, *P* = 0.0001; Additional file 3: Fig. S3a–b). And the increased rate of Iba-1^+^ in MW-exposed mice returned to the normal range at three days after exposure (claustrum, *P* = 0.0004; striatum, *P* = 0.0003; nucleus accumbens, *P* < 0.0001; hippocampus, *P* = 0.0078; Additional file 3: Fig. S3a–b). Notably, there were no differences observed in Iba-1^+^ rate of mice with MW exposure in motor cortex (Bregma + 1.42 to  + 1.70 mm), such as primary motor cortex (M1) (F_(2,12)_ = 0.2236, *P* = 0.8029) and secondary motor cortex (M2) (F_(2,12)_ = 1.289, *P* = 0.3112) (n = 5, Additional file 4: Fig. S4a–c). Hence, our data demonstrated that MW exposure dramatically induced microglial inflammation in cognitive regions in short-term.

### Minocycline attenuates the MW exposure induced microglial activation

**Fig. 4:**
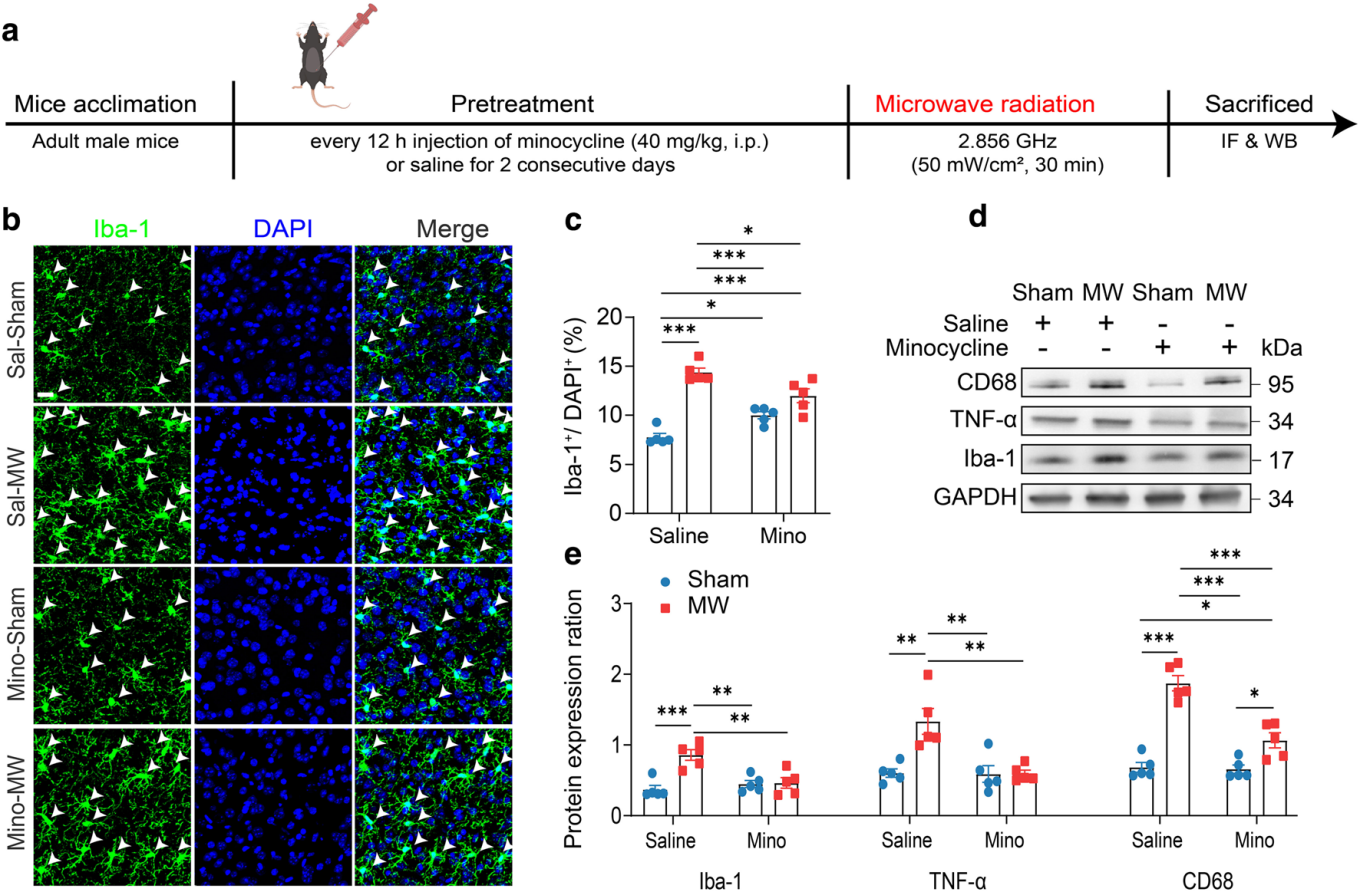
Microglial activation attenuates by minocycline pretreatment. **a** The timeline of experiment design. **b** Representative immunostaining of Iba-1 (green, Alexa Fluor 488) in mPFC. Scale bars: 20 μm. **c** Quantification of Iba-1 positive rate (n = 5). **d**, **e** Representative blot and quantitative analysis of the protein expression of Iba-1, TNF-α and CD68 in mPFC (n = 5). Data are presented as mean ± SEM. *P < 0.05, **P < 0.01, ***P < 0.001 (one-way ANOVA and Tukey’s multiple comparisons test)

Minocycline had been reported to attenuate microglial activation and reduce cognition deficits [13–15]. First, immunofluorescence staining and Weston blot were used to further examine whether minocycline pre-treatment prevent microglial activation of MW exposure (Fig. 4a). Two-way ANOVA analysis (MW × pre-treatment) of Iba-1^+^ rate showed significant difference (interaction effect, F_(1, 16)_ = 21.68, *P* = 0.0003; the main effect of MW, F_(1, 16)_ = 0.8690, *P* = 0.8690; the main effect of pre-treatment, F_(1, 16)_ = 75.41, *P* < 0.0001) (n = 5). And the MW exposed mice with minocycline pre-treated (minocycline-MW) showed no observable difference in Iba-1^+^ rate compared with sham (minocycline-sham) (*P* = 0.0513), yet decreased compared to saline-MW group (*P* = 0.0169), and was found higher than saline-sham group (*P* < 0.0001; Fig. 4b, c).

Moreover, two-way ANOVAs (MW × pre-treatment) of the microglial inflammation-related protein expression revealed similar changes, including the expression of Iba-1 (interaction effect, F_(1, 16)_ = 13.00, *P* = 0.0024; the main effect of MW, F_(1, 16)_ = 5.855, *P* = 0.0278; the main effect of pre-treatment, F_(1, 16)_ = 14.79, *P* = 0.0014), TNF-α (interaction effect, F_(1, 16)_ = 9.937, *P* = 0.0062; the main effect of MW, F_(1, 16)_ = 10.67, *P* = 0.0048; the main effect of pre-treatment, F_(1, 16)_ = 10.24, *P* = 0.0056) and CD68 (interaction effect, F_(1, 16)_ = 19.23, *P* = 0.0005; the main effect of MW, F_(1, 16)_ = 21.80, *P* = 0.0003; the main effect of pre-treatment, F_(1, 16)_ = 80.24, *P* < 0.0001). And Tukey’s multiple comparisons test showed that saline-MW mice was higher than saline-sham (Iba-1, *P* = 0.0005; TNF-α, *P* = 0.0019; CD68, *P* < 0.0001) and minocycline-sham groups (Iba-1, *P* = 0.0036; TNF-α, *P* = 0.0017; CD68, *P* < 0.0001; Fig. 4d, e). While there was no statistical difference between sham and MW exposure by minocycline pre-treatment (Iba-1 and TNF-α, *P* > 0.05; Fig. 4d, e), suggesting minocycline likely attenuate the microglial neuroinflammation caused by MW exposure. Moreover, minocycline attenuated the overexpression of CD36 after MW exposure in PFC, hippocampus and striatum, whereas the downregulation of LRP1 and PPT1 induced by MW were not affected (Additional file 9: Fig S8).

### Minocycline accelerates the recovery of inattention after MW exposure

**Fig. 5:**
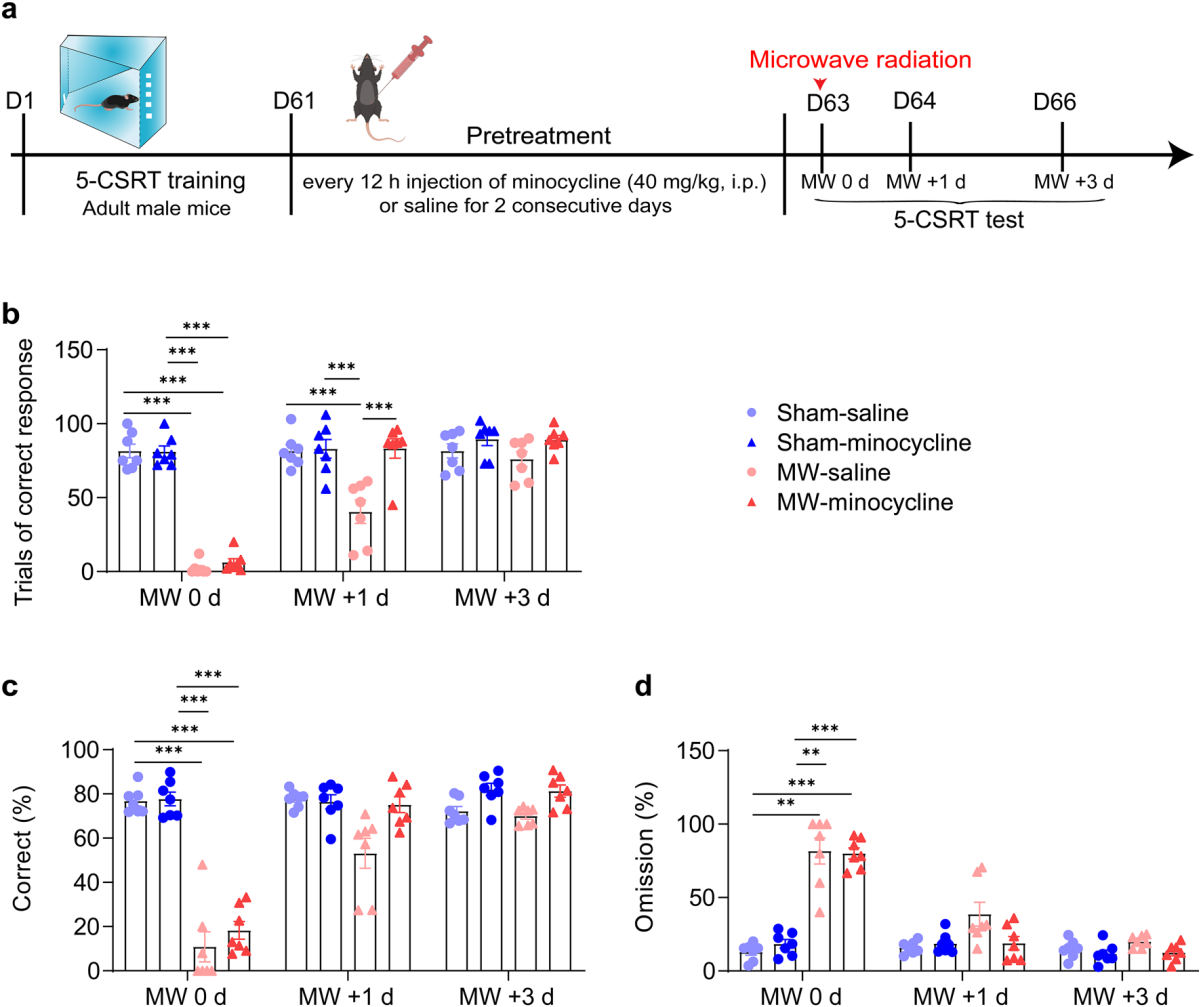
The recovery of inattention induced by MW exposure is accelerated by minocycline. **a** The outline of experiment process. **b**–**d** Quantitative analysis of the trials of correct response, percentage of correct or of omission, respectively (n = 7). Data are presented as mean ± SEM. *P < 0.05, **P < 0.01, ***P < 0.001 (three-way ANOVA and Tukey’s multiple comparisons test)

Next, mice with pre-treatment of normal saline or minocycline were tested by 5-CSRT for attention behavior at 1 h (MW 0 d), 1 day (MW + 1 d), and 3 days (MW + 3 d) after MW (Fig. 5a). Three-way ANOVA analysis (MW × pre-treatment × time) showed difference 5-CSRT performances in mice with different pre-treatment (n = 7, Fig. 5b–d), such as correct trials (interaction of MW × pre-treatment × time, F_(2, 48)_ = 4.127, *P* = 0.0222; interaction of MW × pre-treatment, F_(1, 24)_ = 12.85, *P* = 0.0015; interaction of time × pre-treatment, F_(2, 48)_ = 3.834, *P* = 0.0285; interaction of time × MW, F_(2, 48)_ = 55.63, *P* < 0.0001; the main effect of pre-treatment, F_(1, 24)_ = 23.49, *P* < 0.0001; the main effect of MW, F_(1, 24)_ = 198.8, *P* < 0.0001; the main effect of time, F_(2, 48)_ = 66.78, *P* < 0.0001), percentage of correct (interaction of MW × pre-treatment × time, F_(2, 48)_ = 2.383, *P* = 0.1031; interaction of MW × pre-treatment, F_(1, 24)_ = 5.467, *P* = 0.0280; interaction of time × pre-treatment, F_(2, 48)_ = 0.9371, *P* = 0.3988; interaction of time × MW, F_(2, 48)_ = 76.64, *P* < 0.0001; the main effect of pre-treatment, F(1, 24) = 14.16, *P* = 0.0010; the main effect of MW, F_(1, 24)_ = 134.0, *P* < 0.0001; the main effect of time, F_(2, 48)_ = 75.99, *P* < 0.0001) and percentage of omissions (interaction of MW × pre-treatment × time, F_(2, 48)_ = 1.237, *P* = 0.2992; interaction of MW × pre-treatment, F_(1, 24)_ = 5.627, *P* = 0.0260; interaction of time × pre-treatment, F_(2, 48)_ = 1.519, *P* = 0.2292; interaction of time × MW, F_(2, 48)_ = 59.39, *P* < 0.0001; the main effect of pre-treatment, F_(1, 24)_ = 2.791, *P* = 0.1078; the main effect of MW, F_(1, 24)_ = 123.1, *P* < 0.0001; the main effect of time, F_(2, 48)_ = 63.76, *P* < 0.0001). Further Tukey’s multiple comparisons test revealed that mice with pre-treated saline (Saline-MW group) displayed attention deficits in MW 0 d, including decreased trials of correct response (compare to saline-sham and minocycline-sham group, *P* < 0.0001), percentage of correct (compare to saline-sham, *P* = 0.0007, minocycline-sham group, *P* = 0.0004) and increased percentage of omission (compare to saline-sham, *P* = 0.0036, minocycline-sham group, *P* = 0.0051). And even in MW + 1 d, the correct response still lower in saline-MW mice (compare to saline-sham, *P* = 0.0007, minocycline-sham group, *P* = 0.0004). But, these impairment were recovered in mice pre-treated with minocycline (compare to saline-sham and minocycline-sham group, *P* > 0.9999. compare to saline-MW, *P* < 0.0001). Moreover, 3 days later, all mice exhibited normal attentional behavior (saline-MW group compare to others, *P* > 0.05). Although three-way ANOVA analysis showed statistic difference in interaction and main effect in the percent of accuracy (interaction of MW × pre-treatment × time, F_(2, 48)_ = 3.633, *P* = 0.0339; interaction of MW × pre-treatment, F_(1, 24)_ = 4.807, *P* = 0.0383; interaction of time × pre-treatment, F_(2, 48)_ = 4.547, *P* = 0.0155; interaction of time × MW, F_(2, 48)_ = 6.514, *P* = 0.0031; the main effect of pre-treatment, F_(1, 24)_ = 14.73, *P* = 0.0008; the main effect of MW, F_(1, 24)_ = 9.051, *P* = 0.0061; the main effect of time, F_(2, 48)_ = 5.748, *P* = 0.0208), post hoc analysis suggested no observable difference among groups (all *P* > 0.05; Additional file 2: Fig. S2b).

In addition, mice with minocycline-pretreated alter impulsivity through the trials of premature (interaction of MW × pre-treatment × time, F_(2, 48)_ = 1.781, *P* = 0.1795; interaction of MW × pre-treatment, F_(1, 24)_ = 14.11, *P* = 0.0010; interaction of time × pre-treatment, F_(2, 48)_ = 3.545, *P* = 0.00367; interaction of time × MW, F_(2, 48)_ = 8.752, *P* = 0.0006; the main effect of pre-treatment, F_(1, 24)_ = 5.113, *P* = 0.0331; the main effect of MW, F_(1, 24)_ = 9.279, *P* = 0.0056; the main effect of time, F_(2, 48)_ = 5.264, *P* = 0.0170). Especially in MW 0 d, the saline-sham group is higher impulsivity than minocycline-sham (*P* = 0.0016), saline-MW (*P* = 0.0015) and minocycline-MW group (*P* = 0.0010). But there was no difference in impulsivity between different groups in MW + 1 d and + 3 d (Additional file 2: Fig. S2d).

Therefore, their results demonstrated that the attention behavior quickly recover when mice were pre-treated with minocycline.

### Minocycline dampens the upregulation of NMDAR induced by MW exposure

**Fig. 6:**
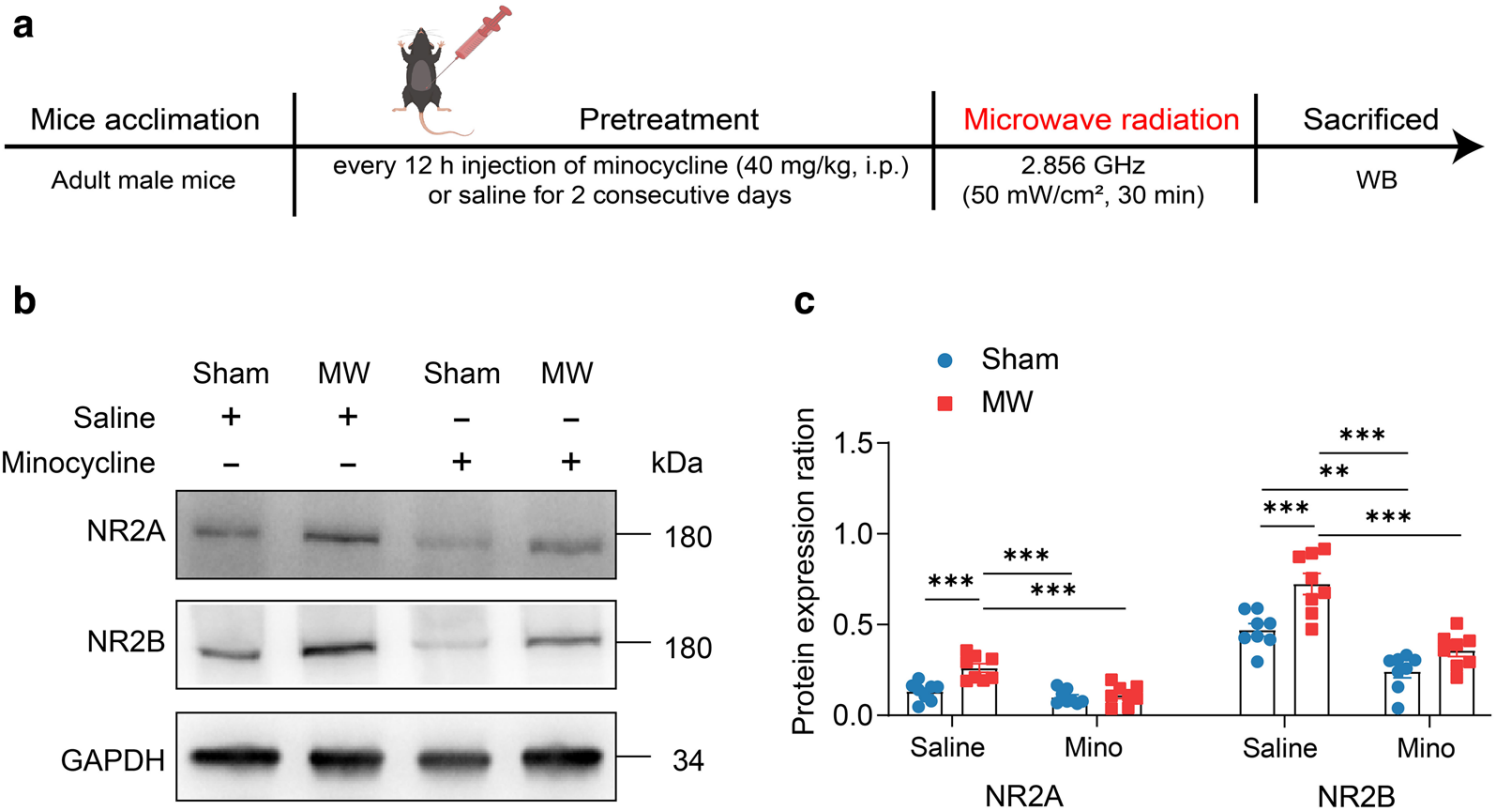
Minocycline inhibits the upregulation of NMDAR induced by MW exposure. **a** The schedule of experiment design. **b**–**c** The protein expression of NR2A and NR2B in mPFC of mice. Data are presented as mean ± SEM (n = 8). *P < 0.05, **P < 0.01, ***P < 0.001 (two-way ANOVA and Tukey’s multiple comparisons test)

To explore the underlying mechanism of attention behavior impaired by MW exposure, we analyzed the protein expression of NMDAR subunits (NR2A and NR2B) in mice mPFC (n = 8, Fig. 6a). At start, two-way ANOVAs (MW × pre-treatment) revealed abnormal changes in the expression of NR2A (interaction effect, F_(1, 28)_ = 9.295, *P* = 0.0050; the main effect of MW, F_(1, 28)_ = 22.24, *P* < 0.0001; the main effect of pre-treatment, F_(1, 28)_ = 13.46, *P* = 0.0010), and NR2B (interaction effect, F_(1, 28)_ = 2.754, *P* = 0.1082; the main effect of MW, F_(1, 28)_ = 51.25, *P* < 0.0001; the main effect of pre-treatment, F_(1, 28)_ = 19.72, *P* = 0.0001). Further multiple comparisons test showed that the expression of NR2A and NR2B was found to be higher in saline-MW mice than those in saline-sham (NR2A, *P* = 0.0003; NR2B, *P* = 0.0001), minocycline-sham (NR2A and NR2B, *P* < 0.0001), and minocycline-MW mice (NR2A and NR2B, both *P* < 0.0001) (Fig. 6b, c), suggesting that minocycline pre-treatment might be able to inhibit the upregulation of NMDAR induced by MW exposure.

## Discussion

**Fig. 7:**
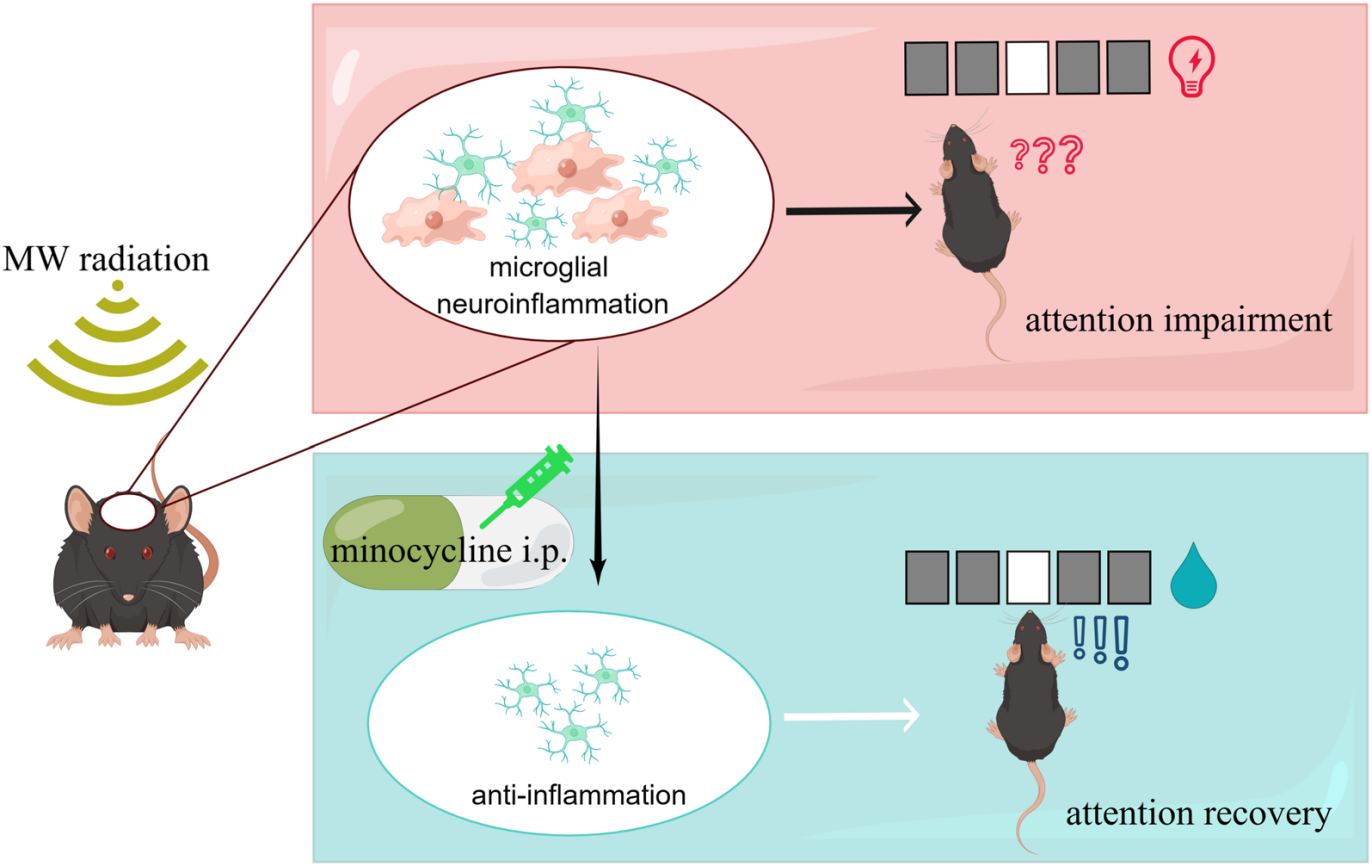
Working model. Microwave radiation induced microglial activation and attention impairment, and minocycline could be used for protection

Our study showed acute attention impairment in mice after MW radiation (a 2.856 GHz radiation source with an average power density of 50 mW/cm^2^ for 30 min) by the 5-CSRT model. And this process was accompanied by microglial activation and the increase of pro-inflammatory cytokine (TNF-α). Moreover, attentional behavior quickly recovered when mice were pre-treated with minocycline, suggesting neuroinflammation might be the potential mechanism of how acute MW exposure alters attention. Furthermore, the NMDAR (NR2A and NR2B) were also increased during the process, and minocycline inhibited the upregulation of NMDAR. Taken together, neuroinflammation and NMDAR might play an important role in the process of MW exposure-induced attention deficits, and minocycline could be effective for protection (Fig. 7).

Since the effect of MW radiation largely relies on its frequency, power density, radiation time and other parameters [12]. Appropriate exposure parameters with rationale are needed to substantiate our study. On the one hand, with regard to the frequency of MW exposure, questionnaire and meta-analysis studies have reported that increased attention problems and the risk of attention-deficit/hyperactivity disorder symptoms are associated with low-frequency MW exposure in daily life, such as television (54–700 MHz), laptop computer (300 Hz–10 MHz) and mobile phone (0.9–2.45 GHz) [1, 36, 37]. Interestingly, Wideband Code-Division Multiple Access (WCDMA, 1.966 GHz) exposure was found to influence vigilance and divided attention in humans (2). However, people who are practitioners in fields related to radar affairs and connected emitters (1–100 GHz) [38], may be radiated and result in compromised health conditions due to high-frequency MW [39]. On the other hand, previous studies mainly explored the long-term effects of MW radiation on learning/memory in rodents by Morris water maze analysis [24, 39], whereas the short-term effects of MW exposure on attention were unclear. Noted that MW-induced cognitive decline was largely affected by its power rather than frequency [40], our study aims to investigate whether the 2.856 GHz MW might lead to an acute alteration of attention with specific average power density at 8, 15, 30 or 50 mW/cm^2^. And we found the attentional deficits revealed by the poor performance during the 5-CSRT task, including decreased correction responses and increased omissions, are associated with the power of MW exposure, mostly effective at an average power density of 50 mW/cm^2^, compared to sham or other densities of MW exposure.

Notably, omission has been viewed as a key parameter indicating attention ability in 5-CSRT [8, 41, 42]. It is shown that omission is the sole factor altered in thought driven top-down attention problems in mice. Moreover, in our study, no significant alterations in accuracy likely excludes learning and cognition deficits. Furthermore, potential motivation change, a key aspect for top-down attention [43], is difficult to be excluded in our model after MW exposure due to the complexity of 5-CSRT performance. However, what we may confirm for the time being is that 50 mW/cm^2^ MW exposure did not affect locomotion in mice, suggesting that the alterations of omission might largely be due to MW-induced attention dysfunction. Remarkably, 15 mW/cm^2^ density seems to increases premature responses but reduces omissions compared to the other densities. While 50 mW/cm^2^ dose increases omissions but reduces premature responses. It makes intuitive sense that mice will not make as many premature responses if they are making a high percent of omissions, but it is interesting that the MW density dictates whether mice are predominantly impulsive or inattentive.

Since we observed obvious short-term attention impairment due to acute MW exposure, we further investigated the mechanisms of this phenotype. Our proteomic analysis suggest microglial activation and cell apoptosis are the most prominent cellular phenotypes after acute MW exposure. Given that microglial activation and neuroinflammation might be druggable targets upstream of cell apoptosis [44, 45], and epidemiology, genetic studies, and systematic review of literature largely suggest that attention-deficit/hyperactivity disorder presents with increased neuroinflammation [46, 47]. We focused on investigating potential microglial activation and neuroinflammation in mice exposed to MW. It is known that neuroinflammation was characterized by four primary features, including elevated levels of pro-inflammatory cytokines, macrophage (microglia) activation, peripheral leukocyte infiltration, and damage to nervous tissue [48]. It has been found that the undesirable activation of microglia severely impairs learning, memory and other essential cognitive functions [49]. Here, the Iba-1 positive rate was increased in regions controlling cognitive functions after MW exposure, including mPFC, claustrum, striatum, nucleus accumbens and hippocampus, while no change in motor cortex such as M1 and M2, likely ruling out a global effect. Next, we showed MW-exposed mice displayed increased Iba-1, TNF-α and CD68, consistent with previous reports that MW radiation promotes the levels of pro-inflammatory cytokines in rodents [12, 50]. In addition to the elevated expression of pro-inflammatory cytokines, microglia in mice with MW exposure show morphological transformation into amoeboid cell shape. Moreover, the anti-inflammation drug pre-treated mice showed faster attentional performance recovery only 1 day after MW exposure, while the control mice went back to normal 3 days later. Hence, microglial inflammation might play an important role in MW-dependent attentional alterations.

Furthermore, we attempted to explore the potential molecular mechanism of attention deficits after MW exposure. Microglia, the main immunocompetent cells in CNS, modulate neuronal activity, facilitate learning, and shape social behavior [51, 52] was found to interact with neurons via their dynamic microenvironmental sensing processes. Several forms of microglia–neuron communication could be promoted by specialized somatic purinergic junctions [52], coactivation of neuronal NMDAR and microglial P2Y12 receptors during seizure [18], and NMDAR triggered ATP-dependent process outgrowth [17]. Noted, that glutamatergic NMDAR plays a central role in PFC activity and its cognitive functions such as working memory, attention and reversal learning [20–23]. In addition, NMDAR is also associated with inflammation [53, 54], and previous study demonstrate a remarkable increase in NMDA (NR2A and C) mRNA expression after viral-like brain inflammation [53]. Here, we observed poor performance of mice in 5-CSRT after MW exposure with increased omissions and decreased correct responses accompanied by elevated expression of NMDAR (NR2A and NR2B). However, other studies report a reduction in NMDAR and long-term impairment of spatial learning and memory after being exposed to the 2.856 GHz pulsed MW source at 50 mW/cm^2^ for 6 min [39], or at 10 mW/cm^2^ for 6 min/day, 5 days/week and up to 6 weeks [24]. Nonetheless, either high or low NMDAR activities are found to be deleterious and will cause apoptosis in neurons [55]. The discrepancy between these studies was likely due to the difference in average power density and radiation time.

Minocycline, an antibiotic with anti-inflammatory properties, has been used as a clinical therapy targeting a number of diseases ranging from atrial fibrillation to Angelman syndrome and schizophrenia [56]. Moreover, studies have demonstrated reduced cognitive deficits due to minocycline [13, 15], while the other indicated minocycline failed to alleviate traumatic brain injury-induced cognitive deficits [57]. The major discrepancies between these studies are the distinct treatment approaches and diverse disease models. Although pre-treated with minocycline was found to accelerate the recovery of inattention induced by MW exposure, it seems to be ineffective on the day of MW radiation, and thus other more effective drugs may be considered. For instance, Flavonoid, a plant-derived compound, was found to display important radioprotective and neuroprotective properties, reducing DNA damage and inflammation in the CNS [38]. In addition to inhibiting microglial activation, minocycline has also been suggested to be a modulator of the NMDAR [58]. Previous studies indicated that the selective blockade of NMDAR located in the mPFC worsens the performance of rats in 5-CSRT task [19], and minocycline reversed the cognitive deficits induced by NMDAR antagonist in animal studies [59]. Here, we found up-regulation NMDAR and abnormal activation of microglia were both inhibited by the pre-treatment of minocycline, and the attention impairment by MW exposure recovered rapidly. Further studies are required to elucidate whether microglial inflammation or NMDAR, is more important in regulating attention impairment after acute MW exposure.

## Conclusion

In summary, we explored the attentional impairment induced by acute MW radiation from the viewpoint of microglial inflammation, which broadens the understanding of the mechanism of MW radiation-induced cognitive deficits. And we found that pre-treated with minocycline accelerates attentional recovery by suppressing abnormal neuroinflammation and NMDAR expression, providing a potential intervention for protecting inattention induced by acute MW exposure. For future studies, a conditional transgenic mouse model specifically activating microglia could be used to validate our findings, and drugs that have better selectivity in terms of mitigating microglial activation could also be applied with an aim to improve attention performance in model organisms exposed to MW.

### Supplementary Information

**Additional file 1 Figure S1**. Locomotor activity of mice in open-field test. **a** The schedule of open field test after MW exposure. **b–c** Total distance and average speed of mice in MW 0 d, MW +1 d and MW +3 d (n =  12, two-way ANOVA analysis). Data are presented as mean ± SEM. **P < 0.01, ***P < 0.001.

**Additional file 2 Figure S2. **Mouse performance in 5-CSRT test. **a** Quantitative analysis of accuracy in mice with diverse MW density exposure (n = 9, two-way ANOVA and Tukey’s multiple comparisons test). **b** Quantitative analysis of accuracy in mice with different pretreatment (n = 7, three-way ANOVA and Tukey’s multiple comparisons test). **c** Quantitative analysis of the premature response in mice with diverse MW density exposure (n = 9, two-way ANOVA and Tukey’s multiple comparisons test). **d** Quantitative analysis of the premature response in mice with different pretreatment (n = 7, three-way ANOVA and Tukey’s multiple comparisons test). Data are presented as mean ± SEM. *P < 0.05, **P < 0.01, ***P < 0.001.

**Additional file 3 Figure S3.** MW exposure induces microglial activation in other regions involved in cognition. **a** Iba-1 (green, Alexa Fluor 488) immunostaining in claustrum, striatum, nucleus accumbens and hippocampus of mice. Scale bars: 50 μm. **b** Quantification of Iba-1 positive rate in four brain subregions after MW exposed. Data are presented as mean ± SEM. **P < 0.01, ***P < 0.001 (n = 5, one-way ANOVA and Tukey’s multiple comparisons test).

**Additional file 4 Figure S4.** No microglial activation in motor cortex in MW-exposed mice. **a** Iba-1 (green, Alexa Fluor 488) immunostaining in M1 and M2 of mice. Scale bars: 25 μm. **b**–**c** Quantification of Iba-1 positive rate in M1 and M2 after MW exposed. Data are presented as mean ± SEM (n = 5, one-way ANOVA analysis).

**Additional file 5 Table S1. **Schedule for stimulus parameters in the 5-CSRT task.

**Additional file 6 Figure S5.** Acute single MW showed a trend of anxiety but did not result in depressive-like behaviors. **a** The schedule of open field test (OFT), elevated plus maze test (EPM) and tail suspension test (TST) after MW exposure. **b**–**c** Time in center of mice during OFT (n = 12). **d**–**e** Time in open/closed arm of mice during EPM (n = 15). **f** Time of freezing/immobile/mobile of mice during TST (n = 15). Data are presented as mean ± SEM. *P < 0.05, **P < 0.01, ***P < 0.001 (two-way ANOVA and Sidak’s multiple comparisons test)

**Additional file 7 Figure S6.** Primary differentially expressed proteins implicated in the activation of microglia and neuroinflammation pathways.

**Additional file 8 Figure S7.** The protein expression of CD36, LRP1 and PPT1 of mice with MW exposure.** a** The schedule of experiment design. **b**–**c** The protein expression of CD36, LRP1 and PPT1 of mice in PFC, hippocampus, striatum (n = 5). Data are presented as mean ± SEM. *P < 0.05, **P < 0.01, ***P < 0.001 (one-way ANOVA and Tukey’s multiple comparisons test).

**Additional file 9 Figure S8.** The protein expression of CD36, LRP1 and PPT1 of mice with minocycline after MW exposed. **a** The schedule of experiment design. **b**–**c** The protein expression of CD36, LRP1 and PPT1 of mice with minocycline in PFC, hippocampus, striatum (n = 5). Data are presented as mean ± SEM. *P < 0.05, **P < 0.01, ***P < 0.001 (two-way ANOVA and Sidak’s multiple comparisons test).

## Data Availability

All relevant data of this study are available from the corresponding authors upon reasonable request.

## Abbreviations

*MW*: Microwave
*5-CSRT*: The five-choice serial reaction time
*CSF*: Cerebrospinal fluid
*LC–MS/MS*: The Liquid Chromatograph Mass Spectrometer
*PFC*: The prefrontal cortex
*NMDAR*: N-methyl-D-aspartic acid receptor
*SAR*: Body special absorption rate
*IF*: Immunofluorescence staining
*WB*: Western blot
*Iba-1*: Ionized calcium-binding adaptor molecule 1
*TNF-α*: Tumor necrosis factor α
*GAPDH*: Glyceraldehyde 3-phosphate dehydrogenase
*DAPI*: 4ʹ,6-Diamidino-2-phenylindole
*SD*: Stimulus duration
*LH*: Limited hold
*ITI*: Inter-trial interval
