# Supplementary figures and images for: Acute exposure of microwave impairs attention process by activating microglial inflammation
Source: Cell Biosci. 2024 Jan 4;14:2. doi: 10.1186/s13578-023-01162-9 (PMC10768366; doi:10.1186/s13578-023-01162-9)

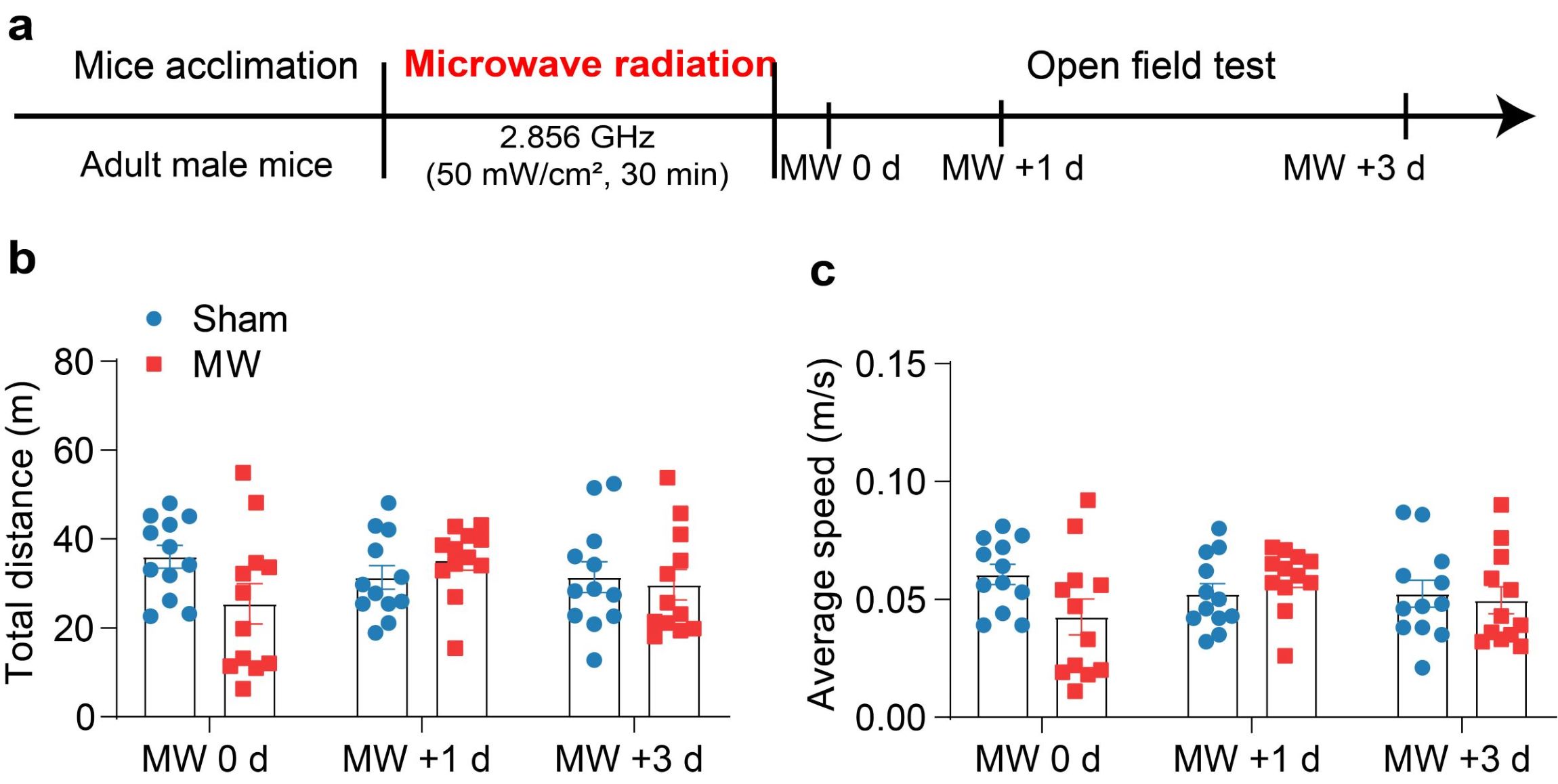

Supplement: Supplementary file 1 — Additional file 1 Figure S1. Locomotor activity of mice in open-field test. a The schedule of open field test after MW exposure. b–c Total distance and average speed of mice in MW 0 d, MW +1 d and MW +3 d (n = 12, two-way ANOVA analysis). Data are presented as mean ± SEM. **P < 0.01, ***P < 0.001. [file 13578_2023_1162_MOESM1_ESM.jpg]

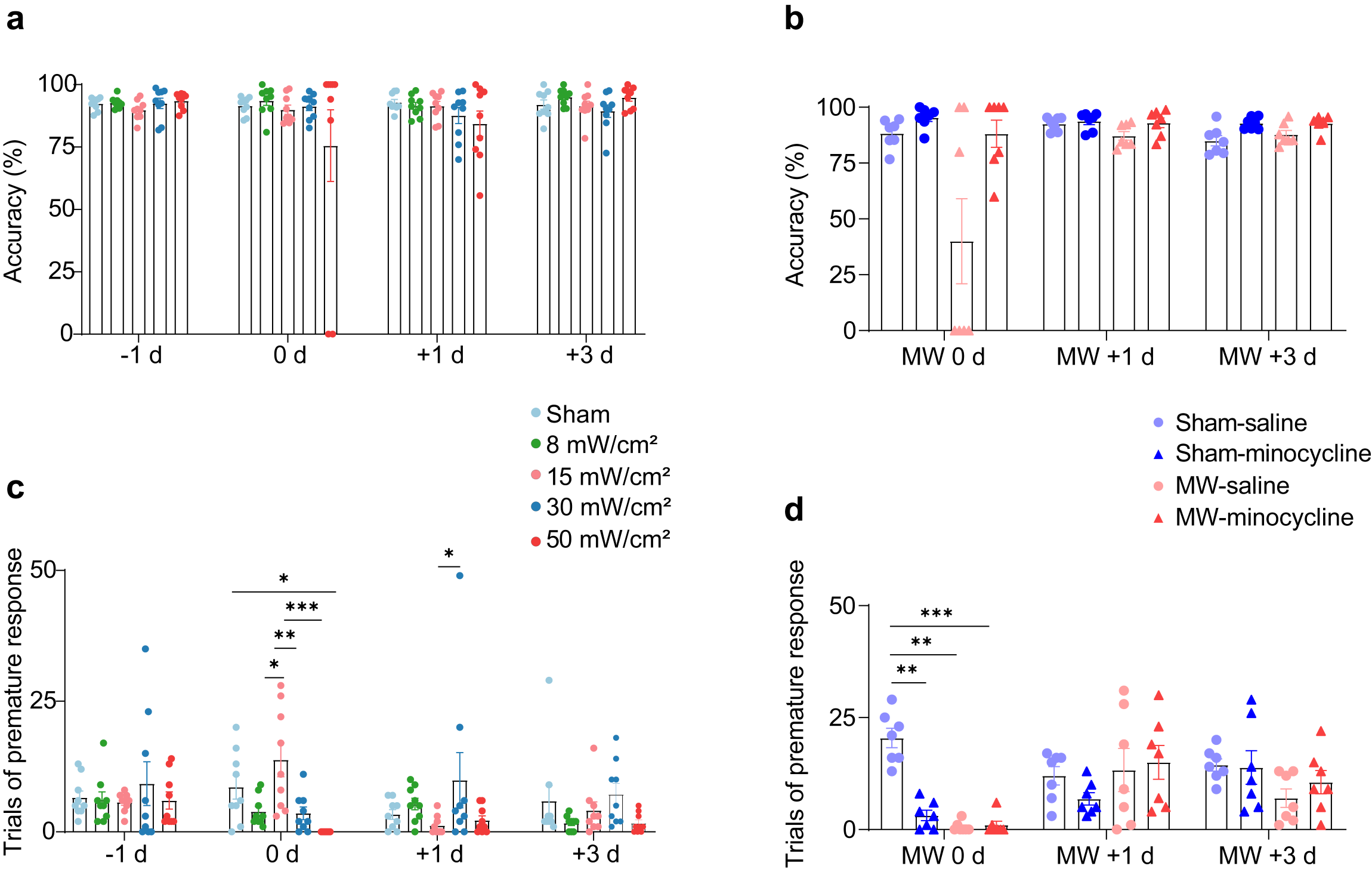

Supplement: Supplementary file 2 — Additional file 2 Figure S2. Mouse performance in 5-CSRT test. a Quantitative analysis of accuracy in mice with diverse MW density exposure (n = 9, two-way ANOVA and Tukey’s multiple comparisons test). b Quantitative analysis of accuracy in mice with different pretreatment (n = 7, three-way ANOVA and Tukey’s multiple comparisons test). c Quantitative analysis of the premature response in mice with diverse MW density exposure (n = 9, two-way ANOVA and Tukey’s multiple comparisons test). d Quantitative analysis of the premature response in mice with different pretreatment (n = 7, three-way ANOVA and Tukey’s multiple comparisons test). Data are presented as mean ± SEM. *P < 0.05, **P < 0.01, ***P < 0.001. [file 13578_2023_1162_MOESM2_ESM.png]

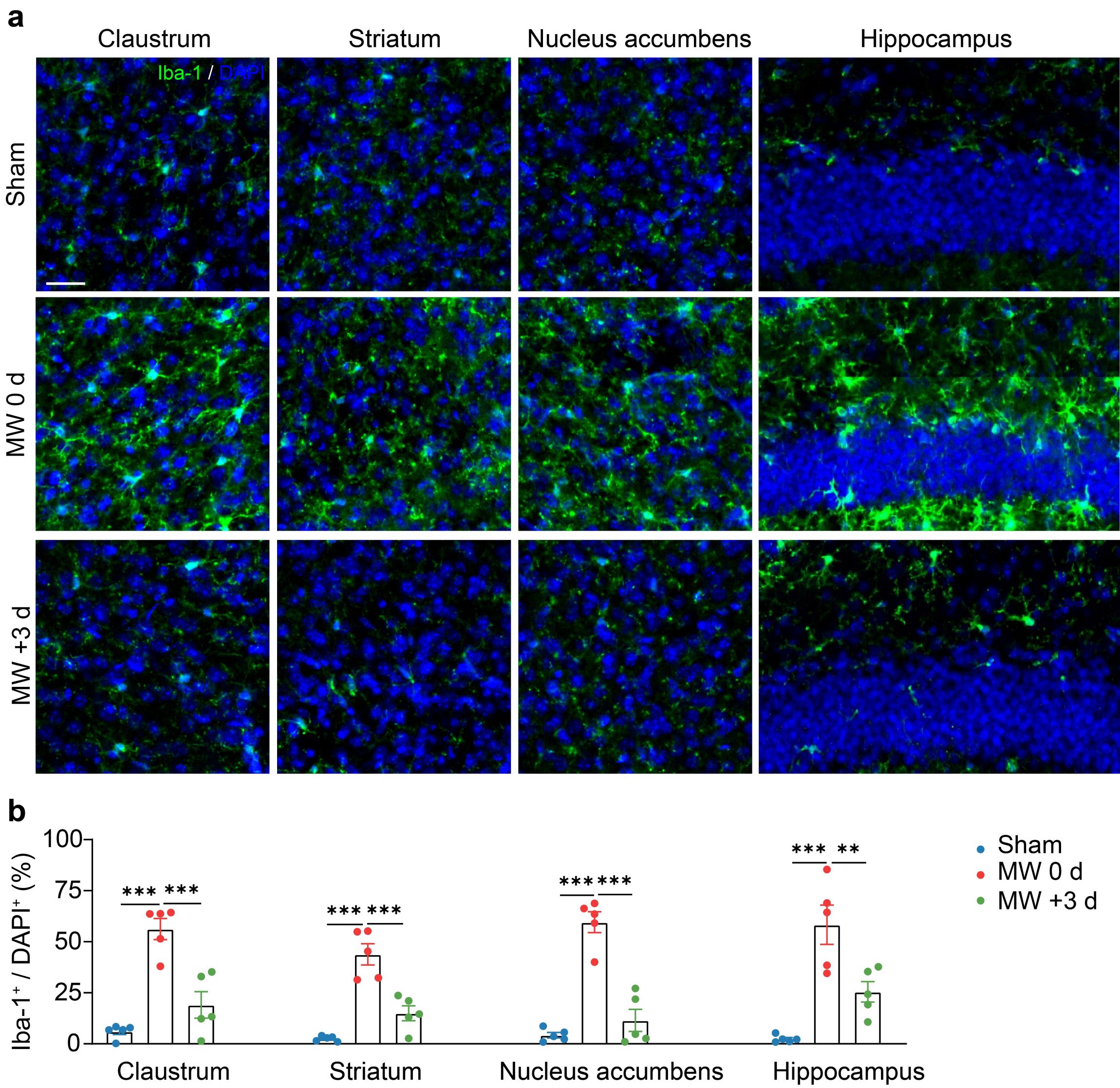

Supplement: Supplementary file 3 — Additional file 3 Figure S3. MW exposure induces microglial activation in other regions involved in cognition. a Iba-1 (green, Alexa Fluor 488) immunostaining in claustrum, striatum, nucleus accumbens and hippocampus of mice. Scale bars: 50 μm. b Quantification of Iba-1 positive rate in four brain subregions after MW exposed. Data are presented as mean ± SEM. **P < 0.01, ***P < 0.001 (n = 5, one-way ANOVA and Tukey’s multiple comparisons test). [file 13578_2023_1162_MOESM3_ESM.jpg]

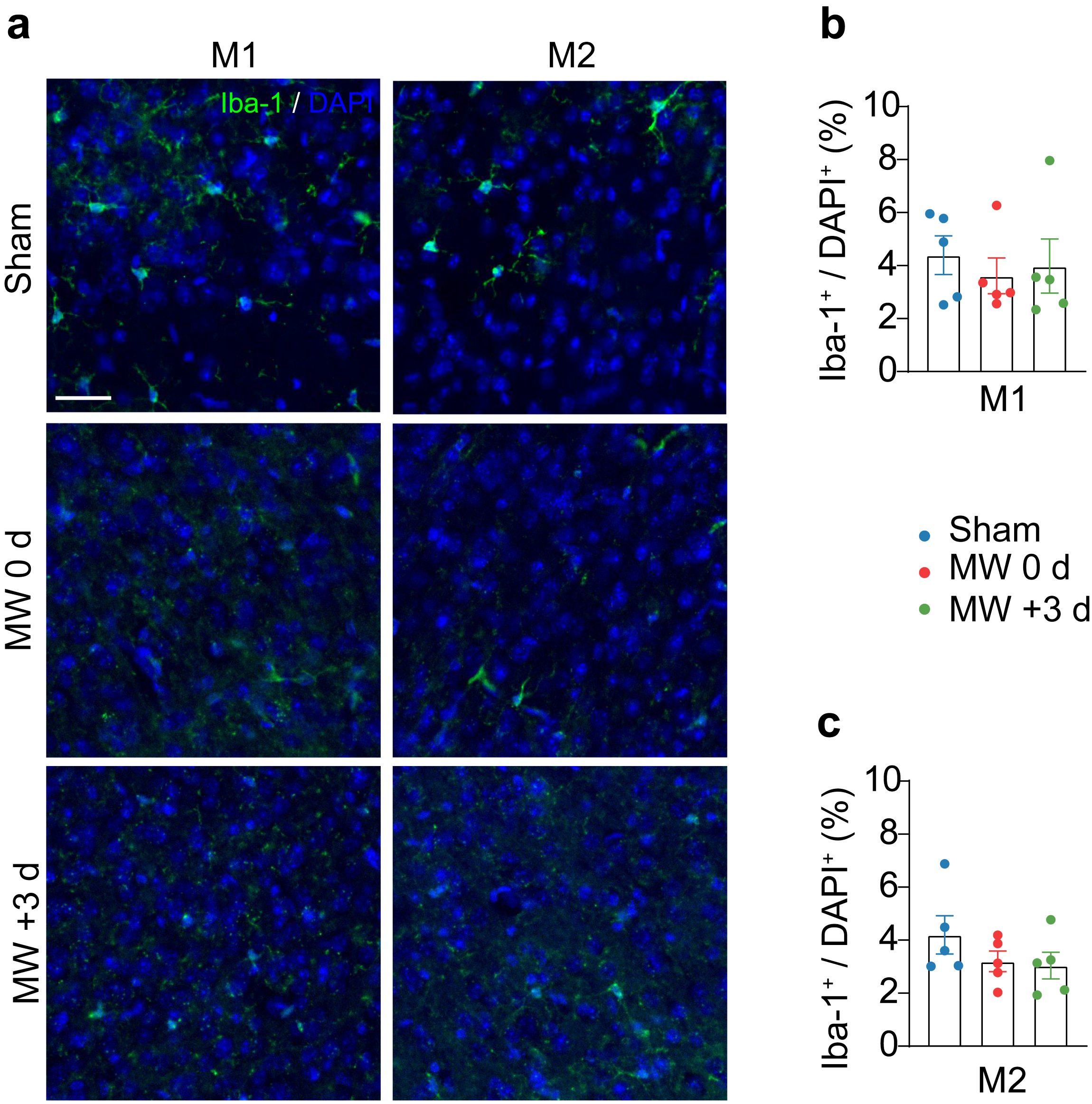

Supplement: Supplementary file 4 — Additional file 4 Figure S4. No microglial activation in motor cortex in MW-exposed mice. a Iba-1 (green, Alexa Fluor 488) immunostaining in M1 and M2 of mice. Scale bars: 25 μm. b–c Quantification of Iba-1 positive rate in M1 and M2 after MW exposed. Data are presented as mean ± SEM (n = 5, one-way ANOVA analysis). [file 13578_2023_1162_MOESM4_ESM.png]

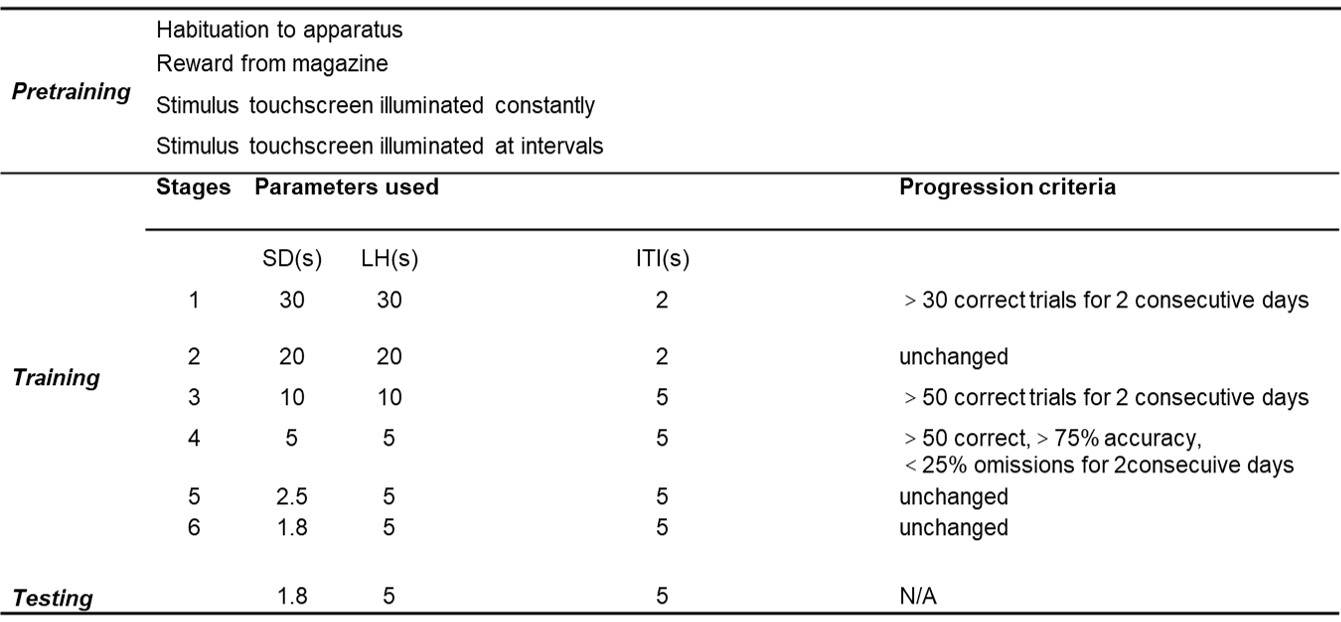

Supplement: Supplementary file 5 — Additional file 5 Table S1. Schedule for stimulus parameters in the 5-CSRT task. [file 13578_2023_1162_MOESM5_ESM.jpg]

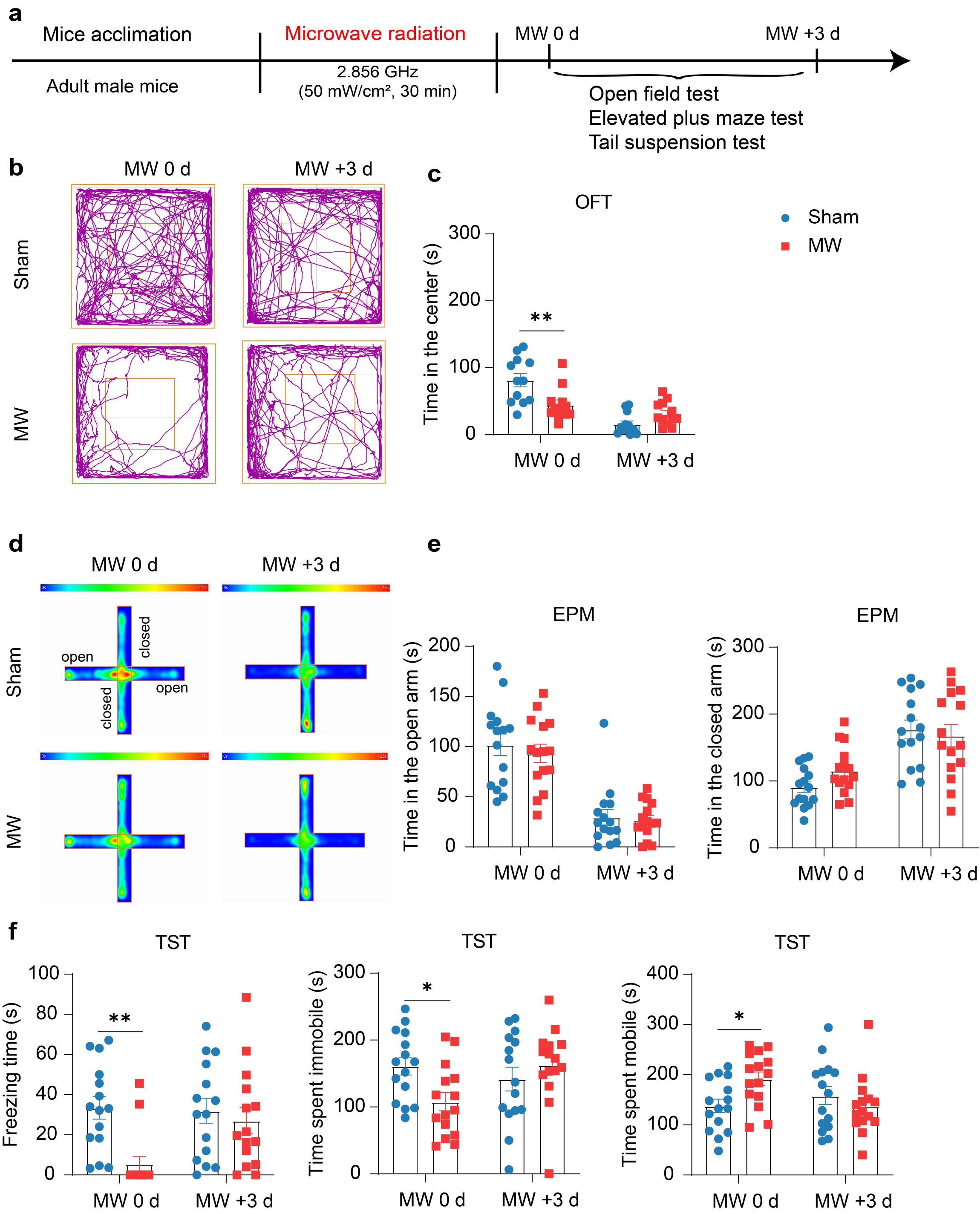

Supplement: Supplementary file 6 — Additional file 6 Figure S5. Acute single MW showed a trend of anxiety but did not result in depressive-like behaviors. a The schedule of open field test (OFT), elevated plus maze test (EPM) and tail suspension test (TST) after MW exposure. b–c Time in center of mice during OFT (n = 12). d–e Time in open/closed arm of mice during EPM (n = 15). f Time of freezing/immobile/mobile of mice during TST (n = 15). Data are presented as mean ± SEM. *P < 0.05, **P < 0.01, ***P < 0.001 (two-way ANOVA and Sidak’s multiple comparisons test) [file 13578_2023_1162_MOESM6_ESM.jpg]

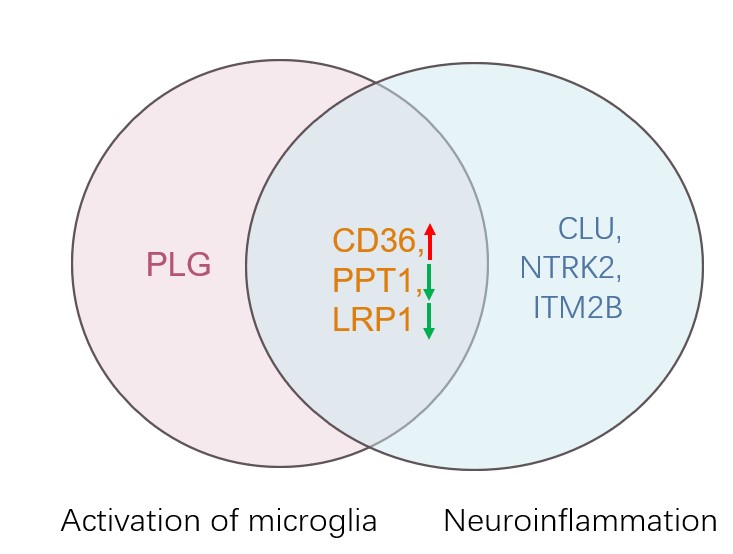

Supplement: Supplementary file 7 — Additional file 7 Figure S6. Primary differentially expressed proteins implicated in the activation of microglia and neuroinflammation pathways. [file 13578_2023_1162_MOESM7_ESM.jpg]

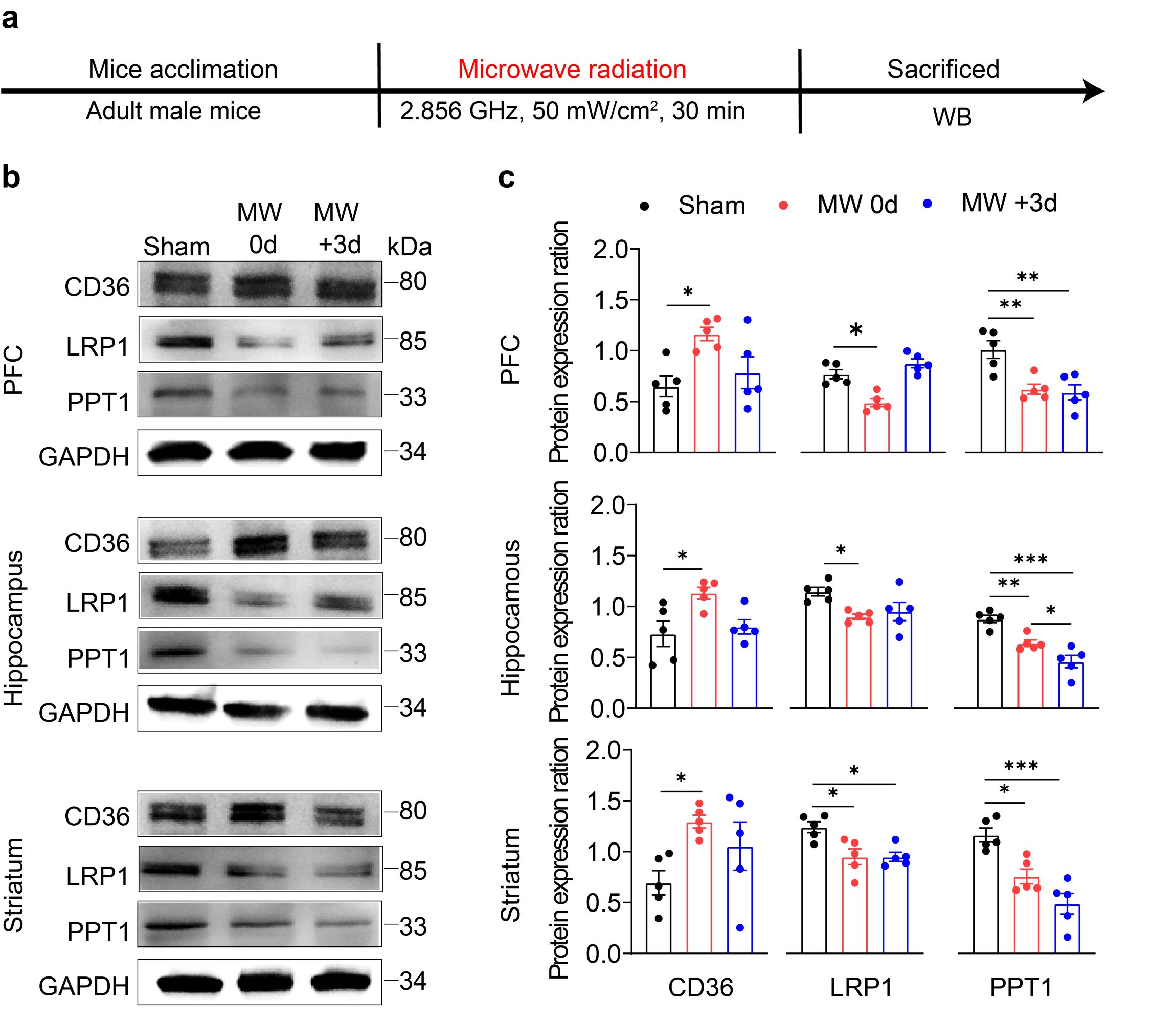

Supplement: Supplementary file 8 — Additional file 8 Figure S7. The protein expression of CD36, LRP1 and PPT1 of mice with MW exposure. a The schedule of experiment design. b–c The protein expression of CD36, LRP1 and PPT1 of mice in PFC, hippocampus, striatum (n = 5). Data are presented as mean ± SEM. *P < 0.05, **P < 0.01, ***P < 0.001 (one-way ANOVA and Tukey’s multiple comparisons test). [file 13578_2023_1162_MOESM8_ESM.jpg]

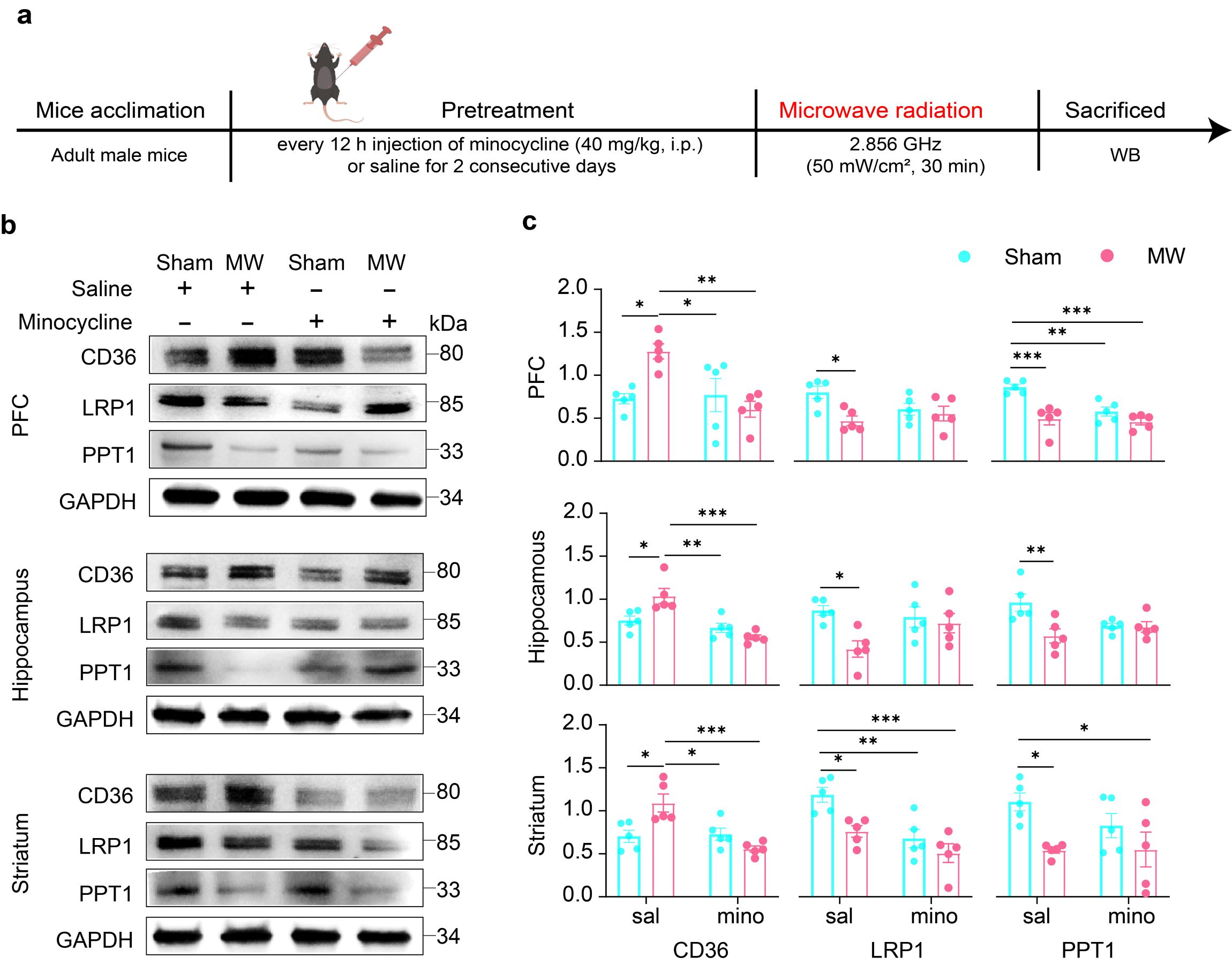

Supplement: Supplementary file 9 — Additional file 9 Figure S8. The protein expression of CD36, LRP1 and PPT1 of mice with minocycline after MW exposed. a The schedule of experiment design. b–c The protein expression of CD36, LRP1 and PPT1 of mice with minocycline in PFC, hippocampus, striatum (n = 5). Data are presented as mean ± SEM. *P < 0.05, **P < 0.01, ***P < 0.001 (two-way ANOVA and Sidak’s multiple comparisons test). [file 13578_2023_1162_MOESM9_ESM.jpg]
